# Effect of crop residues on interception and activity of prosulfocarb, pyroxasulfone, and trifluralin

**DOI:** 10.1371/journal.pone.0208274

**Published:** 2018-12-06

**Authors:** Yaseen Khalil, Ken Flower, Kadambot H. M. Siddique, Phil Ward

**Affiliations:** 1 School of Agriculture and Environment, The University of Western Australia, Perth, WA, Australia; 2 The UWA Institute of Agriculture, The University of Western Australia, Perth, WA, Australia; 3 CSIRO, Wembley, WA, Australia; College of Agricultural Sciences, UNITED STATES

## Abstract

Crop residue retention on the soil surface in no-tillage system can intercept pre-emergent herbicides and reduce their efficacy. Three experiments were conducted to investigate the effect of crop residue amount (0, 1, 2 and 4 t ha^–1^), moisture (wet versus dry), type (wheat, barley, canola, chickpea and lupin) and age (fresh or aged for one year) on the interception and subsequent leaching of prosulfocarb, pyroxasulfone, and trifluralin from the residue into soil. Bioassays, using cucumber and annual ryegrass as indicator plants, were used to assess herbicide activity/availability in the soil and on the residue. Herbicide interception increased considerably as residue quantity increased from 2 to 4 t ha^–1^. After simulated rainfall, which washed herbicide into the soil, complete control of ryegrass occurred for trifluralin with 0 t ha^–1^ residue, for prosulfocarb with 0 and 1 t ha^–1^ residue, and for pyroxasulfone with all residue rates. Therefore, with rain or irrigation, pyroxasulfone was the herbicide least affected by high residue loads. Less chemical leached from the crop residue into the soil after rainfall, when prosulfocarb and trifluralin were applied to wet residue compared with dry residue, but the initial moisture condition had no effect on the leaching of pyroxasulfone from residue. If practically possible, farmers should minimise spraying prosulfocarb and trifluralin onto wet crop residue. Barley and wheat residues intercepted more herbicide than an equivalent mass of canola, chickpea or lupin residue, which was largely due to the increased ground cover with cereal residues. The effect of residue age on herbicide interception and leaching was relatively small and variable. Overall, more herbicide reached the soil when sprayed on one-year old residue than new residue, which was largely due to reduced ground cover with aged residue. A strong positive linear relationship existed between ground cover percentage and growth of bioassay species (r^2^ = 0.75). This means that there was little difference in the ability of residue to adsorb and retain herbicide between crop residue types and ages, such that farmers can simply use the ground cover of the crop residue to assess interception.

## Introduction

Conservation agriculture (CA) is a production system widely adopted around the world, including in Australia. Crop residue retention is one of the pillars of CA, along with zero or minimum mechanical soil disturbance and diverse crop rotations [1–4]. The presence of crop residues on the soil surface protects the soil from erosion, conserves soil moisture, and builds up soil organic carbon (SOC) for crop production [5–8].

Crop residues on the soil surface also play a role in reducing herbicide efficacy in no-tillage (NT) systems [9, 10] because they can intercept a considerable amount of herbicide at the time of application [9, 11, 12]. For example metolachlor activity in the soil declined with high amounts of wheat residue [13]. Even after rainfall, crop residue retained atrazine [11] and metolachlor [13–15]. Crop residues would be expected to reduce the weed control efficacy of herbicides due to interception by crop residue and consequent reduction in the amount of herbicide reaching the soil. However, Prihar et al. [16] found that plots with maize residue had better weed control, than those without maize residue, irrespective of atrazine application. Also, other research showed that soil covered with residue had better weed control with alachlor than uncovered soil [17], but this may be due to the residue smothering the weeds, which compensates for the reduction in herbicide reaching the soil [18]. In addition, Day [19] reported that smothering of weeds by crop residue may weaken weed seedlings, which contributes to increased herbicide efficacy.

Several factors affect whether herbicides are intercepted by crop residues, some being herbicide-related (physio-chemical properties), while others are related to the properties of the crop residues. For example, wet residues have lower sorption capacity for herbicides than the soil underneath [20]. The capacity of a herbicide to volatilise is relatively high for standing straw compared with horizontal straw [20]. As a result, herbicide losses increased when combined with standing, wet residue [20]. However, once the herbicide contacted the soil, the chance of volatilisation decreased due to increased sorption, particularly in soils with more organic matter [20].

Studies conducted by Lal [21] and De-Silva and Cook [22] suggested that 4–6 t ha^–1^ of residue is enough to improve rainfall infiltration and reduce soil erosion. Banks and Robinson [23] reported that the amount of acetochlor, alachlor, and metolachlor detected in the soil progressively declined as the amount of wheat residue on soil the surface increased from 0 to 6.7 t ha^–1^. However, the percent cover may be more closely related to herbicide interception than the amount of residue especially at low residue levels; whereas, amount of residue may be more important with high residue levels (with 100% ground cover), such as in some NT systems. The conversion of residue amounts into coverage for different crops has not been well researched [24].

The type and composition of crop residues also influences the interaction with herbicides. Dao [25] reported that the lignin content of plant stubble might be responsible for most of its sorptive capacity while cellulose, the more abundant plant material, has little impact [25]. Furthermore, older, partially-decomposed straw appears to adsorb more herbicide than fresh straw [20]. This may be due to the decomposition of cellulose and other plant elements, thereby exposing the more reactive lignin compounds [20]. In contrast, Grover [26] found that picloram was not adsorbed on wheat stubble or cellulose but was highly adsorbed on SOM. The physical–chemical mechanisms, such as the sorption of herbicides by lignocellulose may reduce the effective solution concentration, thereby curbing bioavailability and biodegradation [27]. The sorption of chlorimuron and cyanazine increased with the degree of residue decay on hairy vetch (*Vicia villosa* Roth), rye (*Secale cereale* L.) and ryegrass (*Lolium multiflorum* Lam.) residues [28, 29] and was not completely reversible [30].

Common pre-emergent herbicides currently used in NT systems are prosulfocarb, pyroxasulfone and trifluralin [31, 32]. They were introduced to control grasses and small-seeded broadleaves in corn, soybean, sunflower, and field pea in Canada [33–36], United Kingdom [37], United States and Australia [34, 38–40]. As stubble retention is a key component of NT systems, it is important to understand the impact of crop residue amount and type on the activity of these herbicides, especially after rainfall. In this study, prosulfocarb, pyroxasulfone and trifluralin were sprayed onto crop residues followed by simulated rainfall to test the hypotheses that: 1) increased residue amount will intercept more herbicide, which will leach into the soil after rainfall; 2) initially wet residue will leach less herbicide into the soil than initially dry residue; 3) crop residue type will influence the amount of herbicide leached and 4) older residue will leach less herbicide than fresh new residue.

## Materials and methods

Three experiments were conducted to investigate the effect of simulated rainfall on leaching of prosulfocarb (Boxer, 800 g ai L^-1^ (S-benzyl dipropyl(thiocarbamate)), Syngenta Crop Protection AG, Postfach, Switzerland), pyroxasulfone (Sakura, 850 g ai kg^-1^, (5-(difluoromethoxy)-1-methyl-3-(trifluoromethyl) pyrazol-4-ylmethyl 4,5-dihydro-5,5-dimethyl-1,2-oxazol-3-yl sulfone), Bayer Cropscience Pty Ltd, Victoria, Australia) and trifluralin (Treflan, 480 g ai L^-1^, Dow AgroSciences Canada Inc. A Subsidiary of the Dow Chemical Company, Canada) from crop residues into the soil. The amount of herbicide remaining on the crop residue or in the soil was determined by bioassay. Experiment 1 evaluated the effect of the amount of wheat residue on herbicide leaching. Experiment 2 examined the effect of crop residue moisture on the sorption and leaching of these three herbicides and Experiment 3 the effect of crop residue type and age on herbicide leaching.

### Experimental design and management

The three experiments were conducted at The University of Western Australia, School of Agriculture and Environment facilities (–31.9812° S, 115.8199° E). The soil was collected from the surface (0–10 cm) of a farmer’s field near Cunderdin, WA (–31.5844° S, 117.3270° E). The soil was air-dried and passed through a 2-mm sieve prior to analysis (Soil Science Laboratories of The University of Western Australia, Perth and CSBP Soil and Plant Laboratory (www.csbp-fertilisers.com.au). The soil was a sandy loam (74% sand, 12% silt, 14% clay), with pH (CaCl_2_) of 4.4, 2.96 cmol(+)kg^-1^ CEC, exchangeable cations of 2.75 Ca, 0.14 K, 0.45 Mg, 0.07 Na cmol(+)kg^–1^, and 1.8% organic carbon [41]. Four Petri dishes, each containing 50 g of dry soil, were placed onto plastic trays, which were then covered with the crop residue treatment and sprayed with one of three herbicides, except for an unsprayed control. This methods form the basis of all three experiments. The rates of herbicide were 2000 g a.i. ha^–1^ of prosulfocarb, 102 g a.i. ha^–1^ pyroxasulfone and 960 g a.i. ha^–1^ trifluralin, as per the recommended field rates [42]. After spraying, 20 mm of simulated rainfall at 10 mm hr^–1^ was applied, except for the control (nil rainfall) (rainfall simulation details provided in a subsequent section). The crop residues were then air-dried for few hours and ground into small particles using mechanical plant material grinder (www.retsch.com). To minimise sample contamination, the grinder was thoroughly cleaned after each experimental unit with a vacuum and then air compressor to blow air through the grinder. The particle size of the ground residue was determined by sieving 50 g of unsprayed material, with most residues ranging from 2 to 4 mm. The ground residue was placed in plastic bags and stored, along with the Petri dishes of soil, for few days at –20°C until being used in a bioassay to determine bioavailability. The bioassays were conducted with susceptible annual ryegrass (*Lolium multiflorum* Lam., Dargo, Irwin Hunter Seeds, Unit11, 88 Forrest St, Cottesloe, WA 6011, www.irwinhunter.com.au) to detect low herbicide concentrations, and cucumber (*Cucumis sativus* L., Long Green Supermarket), Mr. Fothergill’s Seeds, 15B Walker St, South Windsor NSW 2756, www.mrfothergills.com.au) to detect relatively high concentrations (that kill the ryegrass) [43].

Each experiment had a randomised complete block design with four replications (trays with residue). Experiment 1 compared different amounts of wheat residue from 0, 9.75, 19.5 and 39 g (equivalent to 0, 1, 2 and 4 t ha^–1^). In Experiment 2, 39 g of dry wheat residue (from which 5 g ground residue taken to germinate to bioassay) was applied to each tray (equivalent to 4 t ha^–1^). About 500 ml of water (equivalent to 5 mm of rainfall) was sprayed onto the residue for the wet treatment. In Experiment 3, the equivalent of 4 t ha^–1^ of wheat (*Triticum aestivum* L.), barley (*Hordeum vulgare* L.), canola (*Brassica napus* L.), chickpea (*Cicer arietinum* L.), and lupin (*Lupinus angustifolius* L.) residue were compared at two ages—recent/new (collected a few days after harvest) and aged (one-year-old). Residue amounts were used as treatments, rather than percent ground cover, due to greater accuracy in weighing the different crop residue types, than establishing the same ground cover percentages. Further details are described below.

### Residue details

Different dry crop residues (wheat, barley, canola, chickpea, and lupin) were collected from the same Cunderdin field as the soil and another nearby field (*–*31.641311° S, 117.243087° E), which had different rotations. Two different ages of residue—recent/new collected shortly after harvest, and aged in the field for approximately 10 months (collected before harvest from the residue remaining from the previous season)—were thoroughly mixed by hand (each type and age kept separate) and a representative dry sample was ground and sieved with a 1-mm sieve. A 10 g sample of the residue was sent to Forage Laboratory/One Dairy (www.dairyone.com) for analyses of lignin content, using the ANKOM Technology Method 9 (Method for Determining Acid Detergent Lignin in the Daisy^II^ Incubator) (www.ankom.com). For the lignin analysis, 0.5 g samples were weighed into filter bags and digested for 75 minutes in 2 L of Acid Detergent Fibre (ADF) solution (20 g cetyl trimethyl ammonium bromide (CTAB) to 1L 1.00 N H_2_SO_4_) in an ANKOM A200 Digestion Unit. Samples were rinsed three times with boiling water for 5 minutes in filter bags followed by a 3 minute acetone soak and drying at 105°C for 2 h. The filter bags were then re-weighed after cooling. Following this, the filter bags were covered by 72% w/w H_2_SO_4_ for 3 h in ANKOM Daisy^II^ Incubator at ambient temperature, rinsed with water, dried and weighed as previously described [44].

After applying the residue treatments to the trays, the ground cover percentage was estimated using a digital photograph of the tray and the “Agronomist Panel” option of ASSESS 2.0 Image Analysis Software, [45].

### Herbicide application

The herbicides were applied in a spray cabinet, using a twin-nozzle (50 cm apart) laboratory sprayer fitted with 110° 01 even flat-fan spray jets (Tee jet) delivering herbicide in 117.1 L ha^–1^ of water at 210 kPa, travelling at a speed of 3.6 km h^–1^.

### Rainfall simulation set up and calibration

The rainfall simulator was based on a design by Meyer [46] and Hermsmeier et al.[47]. The structure consisted of a metallic frame shaped in a truncated pyramid (3.10 m × 2.70 m at the base, 3.0 m × 0.26 m at the top, 2.5 m high) built with 32–40 mm diameter tubes of galvanised iron. Four pillar legs supported the rainfall simulator. A laptop computer was used to control a motor at the top of the frame via an RS-232 communication port. The simulated rainfall was applied through three HB1/24–80° VeeJet flat fan nozzles (Spraying Systems, Wheaton, IL) attached to a pipe 2.4 m above the ground that was turned back and forth by the motor through 45°, at a pre-determined rate. Troughs collected water at the end-point of each rotation and returned it to the storage tank. The wait-time of the nozzles at the end-point could be altered to vary the rainfall application rate. A pressure gauge on top of the water inlet manifold was used to regulate and monitor the flow of water to the nozzles. Simulated rainfall was applied at constant rates of 10 mm h^–1^ with a pressure of 80 kPa to apply 20 mm.

### Bioassay conditions

The bioassay conditions were previously reported by Khalil et al. [43]. The experiment was conducted in a 3 m× 4 m growth room. A rack of three shelves equipped with LUMILUX cool white fluorescent lamps (Model L36W/840, OSRAM). Photosynthetically active radiation (PAR) at the top of the plants was 109 μmol m^–2^ s^–1^ (SD ±5 μmol m^–2^ s^–1^) with a 12-h photoperiod. Room air temperature was maintained at 25/22.5°C (SD ±2/1°C) during the light/dark period. Relative humidity in the room was 70% (SD±10%). The bioassay plants, annual ryegrass and cucumber, (5 seeds of each) were planted at 1 cm depth in 9 cm diameter and 1.5 cm deep Petri dishes filled with either soil or ground wheat residue. The plants were hand watered on a daily basis by adjusting the media moisture to near field capacity with fresh deionized water. Field capacity for both media was defined following the procedures of Somasegaran and Hoben [48], using a 250 ml plastic cylinder with hole on the bottom. The cylinder was filled with samples (soil or ground residue), then tap water was poured into the cylinder wetting two third of the volume of the media in the cylinder. The column was allowed to equilibrate in the laboratory for 48 hours. Then samples were oven dried at 110°C to constant weight and percent moisture in the samples calculated. Shoot length of each plant was measured 7 d after sowing, following removal from the Petri dishes and washing away the soil or crop residue with running tap water. Shoot length as a percentage of the untreated control (UTC) was calculated for each media using the formula [*Shoot length (% of untreated control) = L*_*t*_ *× (100/L*_*0*_*)*] where L_t_ is the shoot length measured in the herbicide-treated soil or wheat residue and L_0_ is the shoot length in the untreated soil or wheat residue.

### Data analysis

Each combination of herbicide (prosulfocarb, pyroxasulfone and trifluralin), bioassay medium (residue and soil) and bioassay species (ryegrass and cucumber) was analysed separately; the ryegrass and cucumber data are both shown, as in some instances no ryegrass germinated. The data were tested for normality and homogeneity of variance before conducting ANOVA on the shoot length data, using GenStat 12 [49], to test for significance at P ≤0.05.

In Experiment 1 a two-way ANOVA was performed to compare the different amounts of wheat residue × (+/-) rainfall. In Experiment 2, a one-way ANOVA was conducted to compare the three treatments [dry residue, wet residue, and treated control (sprayed with herbicide but no rainfall applied)]. In Experiment 3, a three-way ANOVA was performed (crop residue type × crop residue age × (+/-) rainfall).

A series of linear regression analyses for percent ground cover against cucumber shoot length (% of untreated control, as a measure of herbicide activity) in the soil was conducted. The cucumber bioassay was used as no ryegrass germinated for many of the treatments, due to relatively high herbicide concentrations. Firstly, a regression of the cucumber (in soil) data without simulated rainfall was performed, followed by a regression where the data were classified (grouped) by herbicide. Regressions were then undertaken separately for each herbicide with the data classified by rainfall (vs nil rainfall).

## Results and discussion

### Experiment 1—Residue amount

**Fig 1:**
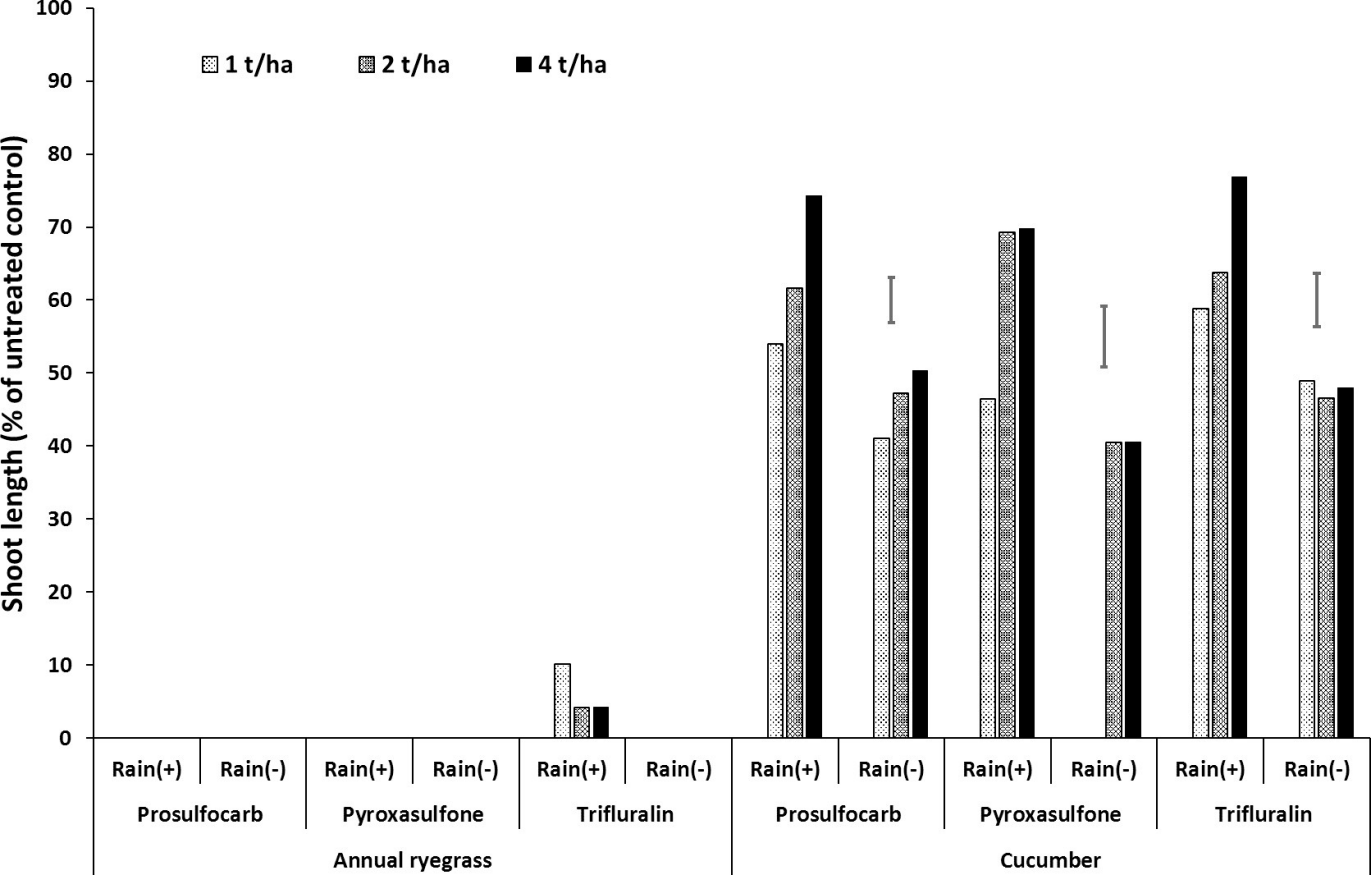
Effect of wheat residue rate (0–4 t ha^–1^) on shoot length (% of untreated control) of annual ryegrass and cucumber grown in wheat residue (note: 0 t ha^–1^ had no residue in the bioassay) as a bioassay media, after spraying prosulfocarb, pyroxasulfone and trifluralin followed by 20 mm (+) or nil (–) rainfall. Bars show LSD at P = 0.05 for comparisons within the same bioassay species.

Annual ryegrass did not germinate in the wheat residue sprayed with prosulfocarb and pyroxasulfone, even after leaching with simulated rainfall. Annual ryegrass germination was <10% of the untreated control for trifluralin after rainfall (Fig 1, S1 Table, S1 Text). Overall, shoot length of cucumber in wheat residue generally increased with residue amount, indicating a dilution of the herbicide as residue amounts increased. Shoot length of cucumber grown in the residue medium declined in the absence of leaching rainfall, due to higher concentrations of herbicide remaining on the residue.

**Fig 2:**
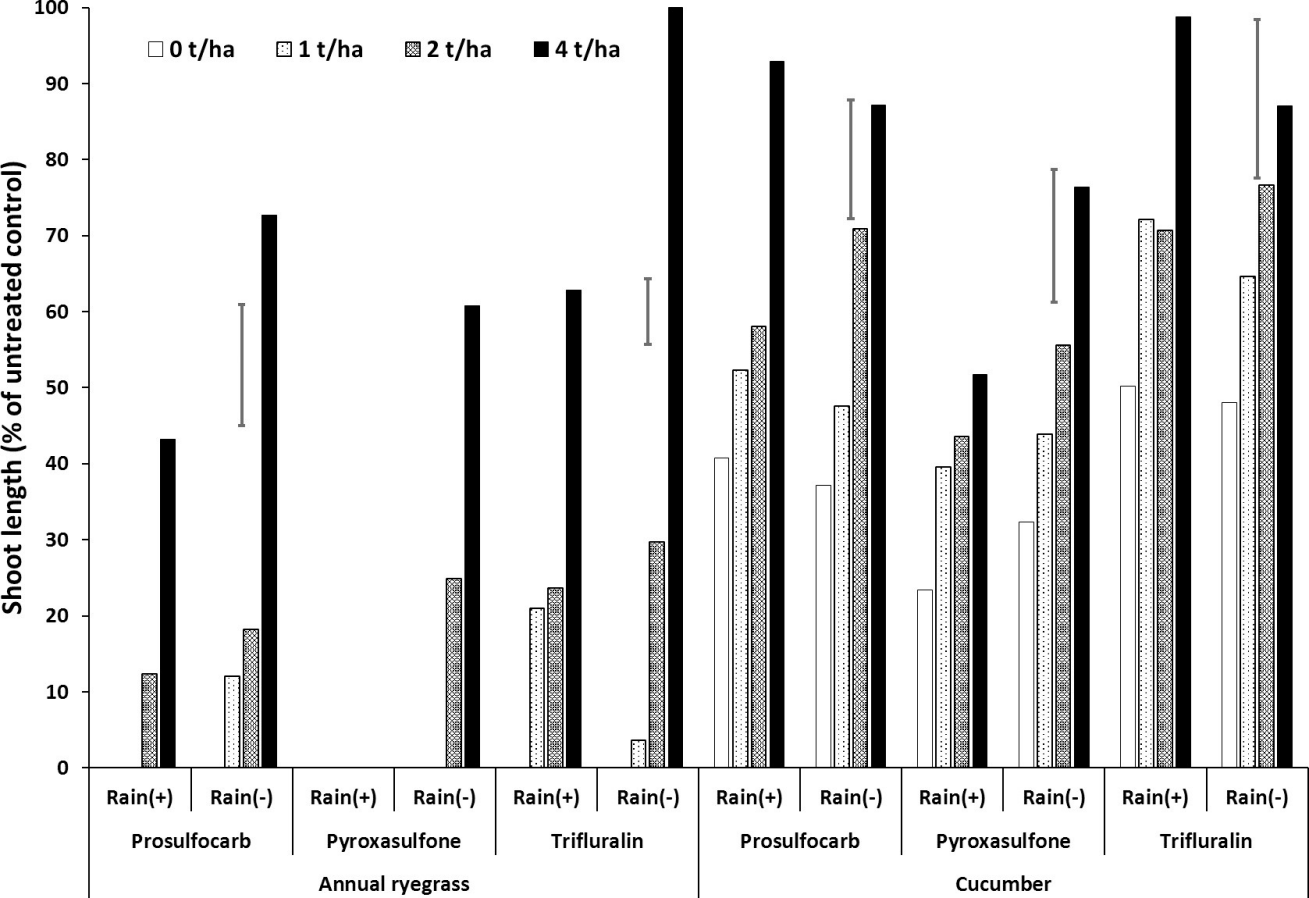
Effect of wheat residue rate (0–4 t ha^–1^) on shoot length (% of untreated control) of annual ryegrass and cucumber grown in soil as a bioassay media, after spraying prosulfocarb, pyroxasulfone and trifluralin followed by 20 mm (+) or nil (–) rainfall. Bars show LSD at P = 0.05 for comparisons within the same bioassay species.

In the absence of simulated rainfall, the growth of the bioassay species in the soil increased with increasing amount of residue, with a marked increase in shoot length from 2 to 4 t ha^–1^ (Fig 2, S1 Table, S1 Text). Rainfall, which leached herbicide from the residue into the soil, generally reduced ryegrass shoot length in the soil for all residue rates, but especially at 4 t ha^–1^ (Fig 2, S1 Table, S1 Text). Prosulfocarb killed all the ryegrass in the soil after rainfall at 0 and 1 t ha^–1^ of residue, whereas pyroxasulfone killed all the ryegrass even at 4 t ha^–1^ of residue. Trifluralin only achieved 100% kill at 0 t ha^–1^ of residue, but there was >70% shoot inhibition up to 2 t ha^–-1^ residue. The cucumber bioassay in soil was much less sensitive, and rainfall only reduced shoot length with pyroxasulfone (Figs 1 and 2, S1 Table, S1 Text).

Overall, rainfall improved the weed control efficacy of each herbicide when sprayed into fields with high residue levels, especially for pyroxasulfone. This suggests that, of the three herbicides, pyroxasulfone should be considered by applicators as a pre-emergent herbicide when the amount of residue on the soil surface exceeds about 2 t ha^–1^, as rainfall can easily wash the herbicide into the soil, but for residue amounts less than 2 t ha^–1^, prosulfocarb may also be effective. Trifluralin should be considered when it can be incorporated into the soil either by rainfall immediately after application or with seeding machinery. Reduced amounts of herbicide reaching the soil are due to interception by crop residue [9, 11, 12, 50]. Banks and Robinson [13] reported that increased levels of wheat residue on the soil surface decreased the amount of acetochlor, alachlor and metolachlor in the soil, which reduced weed control and that 13 mm of subsequent irrigation washed 15–20% of the original herbicide application into the soil. Also, the residue retained more metolachlor than acetochlor or alachlor. Other studies have shown that plant residues do not significantly reduce weed control efficacy. For example, Crutchfield, Wicks [18] found that wheat straw levels up to 6.8 t ha^–1^ did not reduce weed control efficacy of metolachlor applied at 1.5 times the recommended rate, as the straw itself provided some measure of weed control and compensated for less herbicide reaching the soil.

In our study, the physical effect of crop residues on weed suppression could not be tested in the bioassay. In other research, the efficacy of a mixture of alachlor and atrazine in controlling weeds was not affected by surface mulching when recommended rates of herbicide were used [51]. Similarly, Erbach and Lovely [50] reported that 6.2 t ha^–1^ of non-chopped corn stems did not reduce the effectiveness of weed control when using recommended rates of atrazine or alachlor, but did when lower herbicide rates were used. Presumably, chopping the straw would increase the proportion of ground covered by the residue, which would reduce weed control efficacy. Therefore, a key factor when using herbicides with high levels of crop residue is to ensure that sufficiently high rates of the chemical are used along with high carrier/spray volumes, as shown by Borger, Riethmuller [52]. These authors found that increasing the carrier volume from 30 to 150 L ha^–1^ improved the average control of ryegrass by trifluralin and pyroxasulfone from 53 to 78%. Further research should be conducted to assess optimal rates for the different pre-emergent herbicides to achieve good weed control at different crop residue levels.

### Experiment 2 –Residue moisture

**Fig 3:**
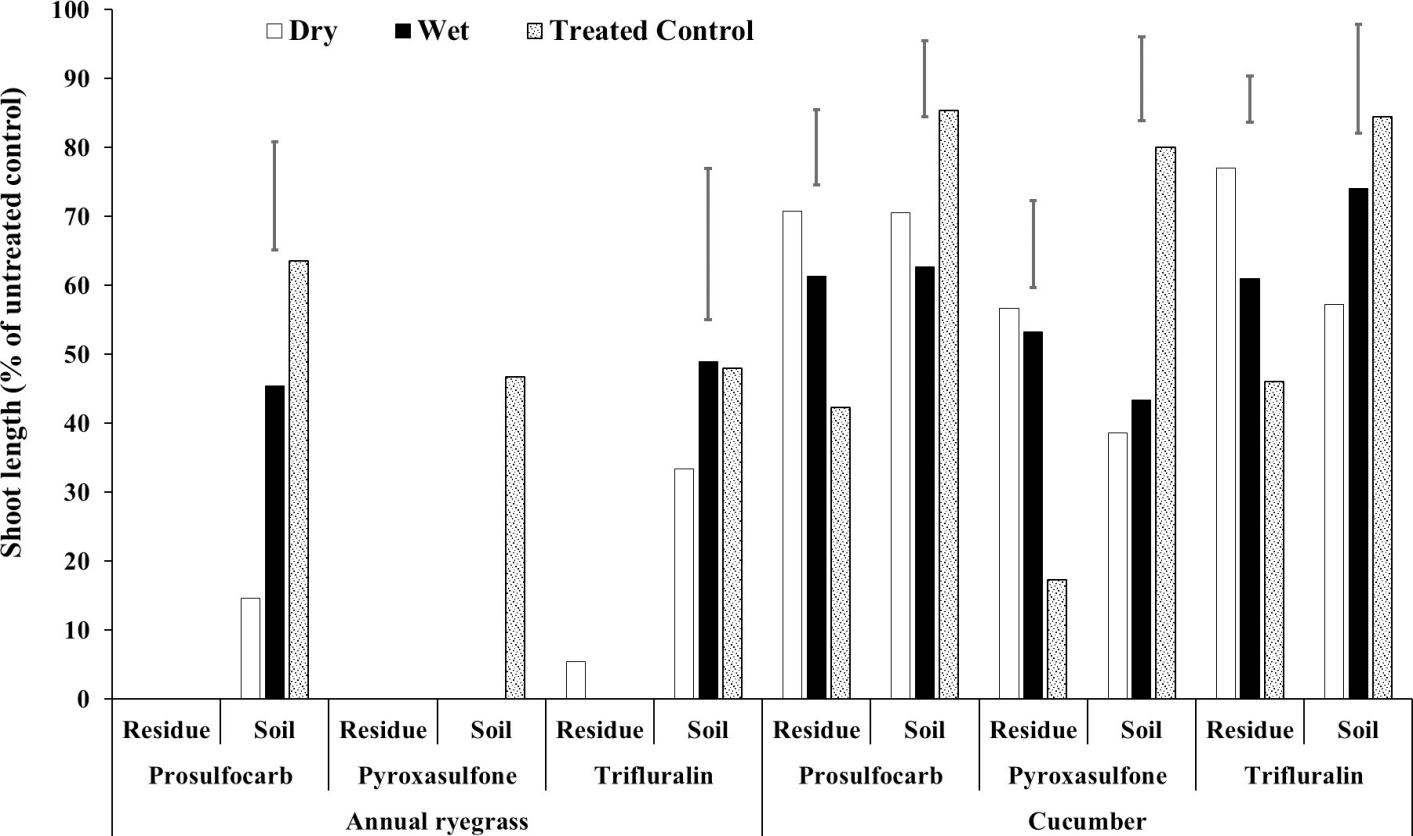
Effect of crop residue moisture on shoot length (% of untreated control) of annual ryegrass and cucumber grown in wheat residue and soil bioassay media, after spraying prosulfocarb, pyroxasulfone and trifluralin followed by 20 mm of rainfall. Bars show LSD at P = 0.05 for comparisons within the same bioassay species, herbicide and media.

The presence of residue increased shoot length compared with the control (Fig 3, S2 Table, S2 Text). The differences in herbicide leaching between wet and dry residue were not always clear-cut. Virtually no ryegrass germinated with any of the herbicides in the residue bioassay, indicating that phytotoxic levels remained on the residue even after rainfall. The cucumber data showed that rainfall leached some herbicide from the residue relative to the treated control (with no rainfall applied) and that less herbicide leached with initially wet than dry residue, although no significant differences were evident for pyroxasulfone. The same trend occurred for the soil bioassays, where less herbicide tended to reach the soil with wet than dry residue, although again no significant differences were evident for pyroxasulfone. These results indicate that in an NT system, with crop residue covering the soil, more prosulfocarb and trifluralin reaches the soil after rainfall when the chemicals are sprayed onto dry rather than wet residue. The moisture status of the residue has little effect on the amount of pyroxasulfone that reaches the soil after subsequent rainfall. From a practical point of view and to minimise the loss of intercepted herbicides, NT farmers should avoid spraying prosulfocarb and trifluralin onto wet crop residue otherwise they may need to increase the herbicide application rate to compensate for losses when sprayed onto wet residues. Heavy dew that wet the residue, may also have a similar effect in reducing the efficacy of these herbicides, if the residue was still wet when sprayed. Erbach and Lovely [50] reported that wetting corn residue before or after alachlor application had no significant effect on foxtail millet control.

### Experiment 3—Residue type and age

The ability of crop residue to adsorb and retain intercepted herbicide is related to its lignin concentration [25]. Older residues (left in the field for a year) of chickpea, canola, and lupin had higher lignin concentrations than new residues (collected shortly after harvest). In contrast, the lignin concentration of cereal residues (wheat and barley) was similar or slightly lower in the aged compared with the recently collected material (Table 1), which is likely due to the relatively slow decomposition of cereals, as shown by Reinertsen, Elliott [53].

**Table 1:** Dry matter and lignin concentration for different residue types and ages.

Crop residue type	Age	Dry matter (%)	Lignin (% DM)
Chickpea	Aged✦	93.4	18.8
Chickpea	Recent^✶^	92.4	12.9
Canola	Aged	93.3	16.1
Canola	Recent	93.6	12
Lupin	Aged	92.2	14.3
Lupin	Recent	91.1	10
Wheat	Aged	92.2	6.8
Wheat	Recent	92.8	9.1
Barley	Aged	92.6	6.6
Barley	Recent	93.1	7.1

^*✶*^ Recent residue collected shortly after the harvest

✦Aged residue left in the field for a year Ground cover percentage varied with both residue type and age (Table 2).

Aged residue generally had less ground cover on a mass basis than fresh residue for canola, chickpea and lupin, whereas the differences were relatively small for cereal residues. Lupin stubble had the least ground cover, followed by chickpea, canola and the cereals, which had >90% ground cover for the 4 t ha^–1^ of residue applied. Leonard [54] reported that 50% ground cover was achieved with 1 t ha^–1^ cereal residue, 2 t ha^–1^ lupin residue and 3 t ha^–1^ of canola residue. Leonard [54] and Leys and Heinjus [55] reported 100% ground cover for wheat residue and 80% for lupin residue at 6 t ha^–1^ (standing and flat) in the field. Therefore, it would be expected that lupin, chickpea and canola residue would have less impact on herbicide efficacy than an equivalent amount of cereal residue.

**Table 2:** Ground cover percentage of different crop residue types and ages estimated by ASSESS 2.0 [45].

	Crop residue type					
		Barley	Wheat	Canola	Chickpea	Lupin
**Crop residue age**	Recent	100	99	92	63	44
	Aged	99	91	42	45	27

### Herbicide remaining on the residue

**Fig 4:**
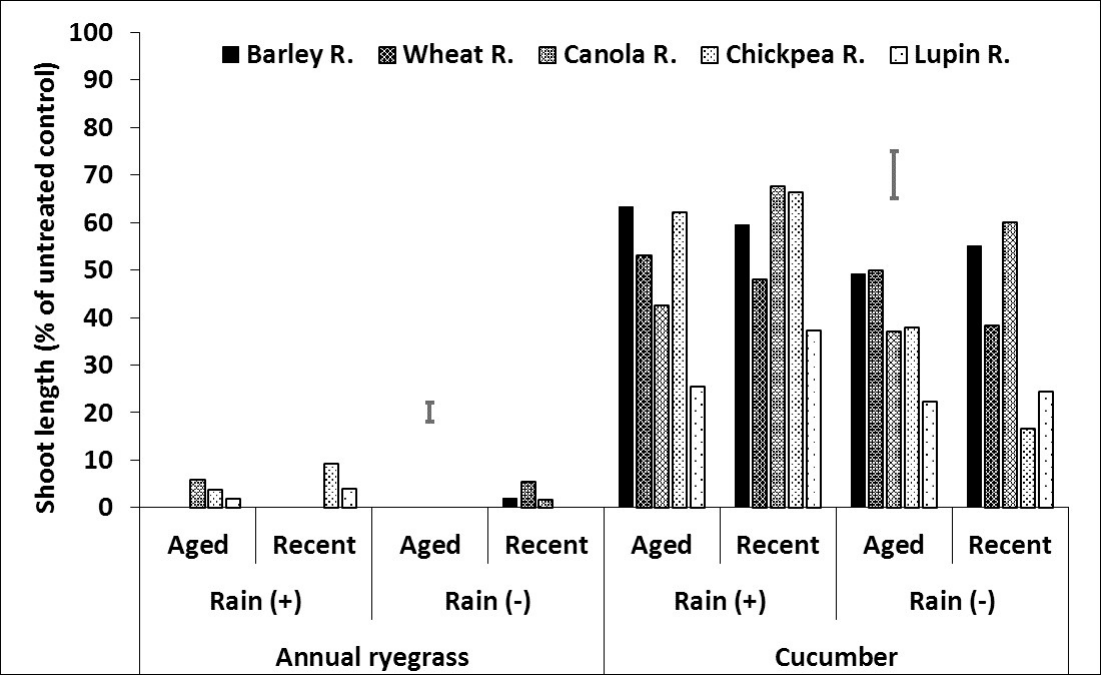
Effect of crop residue type and age on shoot length (% of untreated control) of ryegrass and cucumber in crop residue, after spraying with prosulfocarb followed by 20mm (+) or nil (–) rainfall. LSD bars show interactions between rainfall and age of residue at P = 0.05 for comparisons within the same bioassay species.

**Fig 5:**
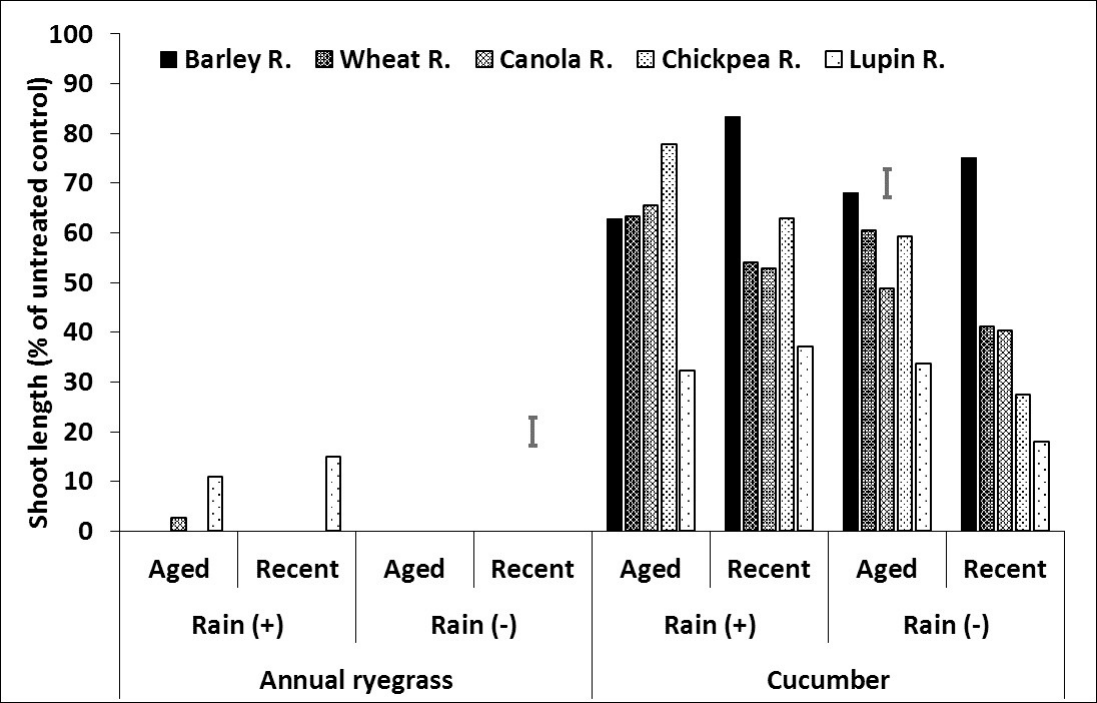
Effect of crop residue type and age on shoot length (% of untreated control) of ryegrass and cucumber in crop residue, after spraying with pyroxasulfone followed by 20mm (+) or nil (–) rainfall. LSD bars show interactions between rainfall and age of residue at P = 0.05 for comparisons within the same bioassay species.

**Fig 6:**
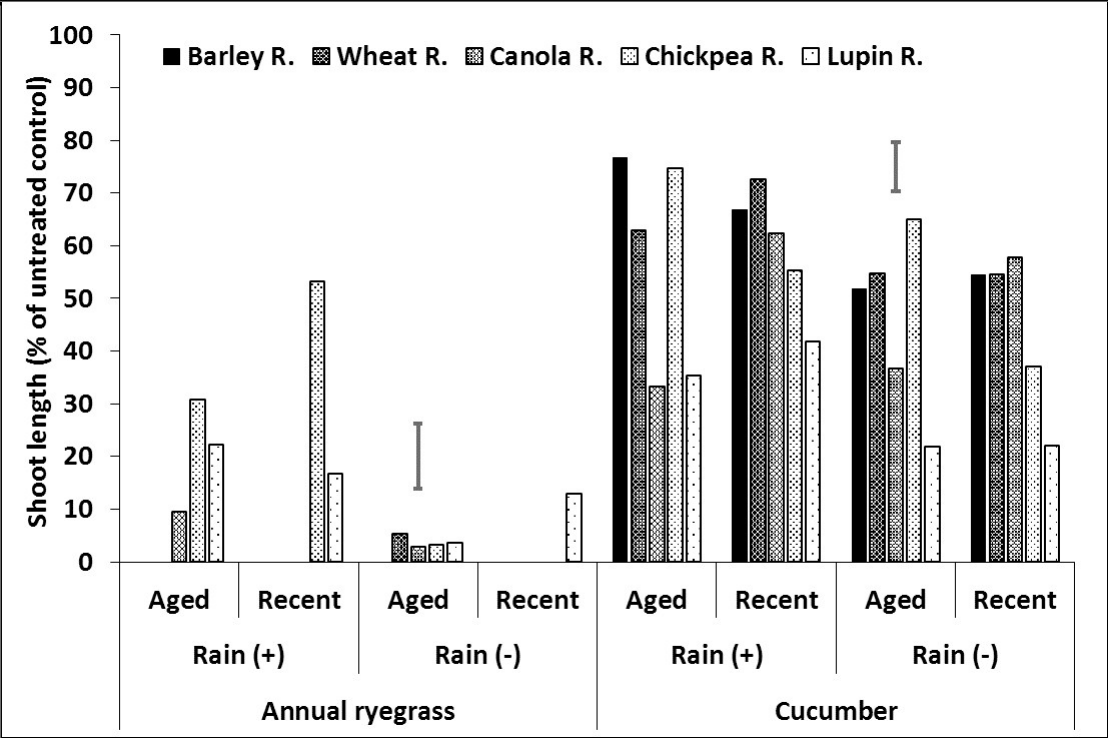
Effect of crop residue type and age on shoot length (% of untreated control) of ryegrass and cucumber in crop residue, after spraying with trifluralin followed by 20mm (+) or nil (–) rainfall. LSD bars show interactions between rainfall and age of residue at P = 0.05 for comparisons within the same bioassay species.

Ryegrass shoot length declined in the crop residue medium and, in some instances plants were killed, by the relatively high concentration of herbicide retained in the crop residues, especially with no rainfall (Figs 4–6, S3 Table, S3 Text). As a result, the focus was on the cucumber bioassay results.

For prosulfocarb remaining in the residue, there was an interaction between residue type, age and rainfall (Fig 4, S3 Table, S3 Text). Overall, barley and wheat residues produced the longest cucumber shoot lengths and lupin the shortest, indicating highest concentrations of ‘bioavailable’ prosulfocarb were retained in lupin residue. Martin et al. [56] reported that corn residue (6–8.9 t ha^-1^) retained 50% and 30% of tested herbicides (alachlor, atrazine, cyanazine and propachlor) after applying 10 mm and 35 mm of water, respectively. Dang et al. [57] also reported that sugarcane residue (5 t ha^-1^) retained 20% of sprayed herbicides (atrazine, ametryn, diuron, and hexazinone) after 30 mm of rainfall. This indicates that different amounts of sprayed herbicides can be retained by different crop residues. In this study, the canola and chickpea results were variable. In the absence of rainfall, aged wheat and chickpea residue had greater shoot length than the equivalent new residue, and the reverse occurred with canola. Age of material had little effect on prosulfocarb activity in barley and lupin residue. Rainfall increased cucumber growth the most with chickpea residue, for both aged and new material, but only had a small effect on cucumber shoot length with barley residue and an intermediate with wheat and canola residues. Rainfall increased shoot length in new lupin residue but had little effect on aged lupin residue.

For pyroxasulfone remaining in the residue, cucumber shoot length followed a similar pattern to prosulfocarb where barley had the greatest shoot length in most instances, particularly with new residue, followed by wheat, canola and chickpea (Fig 5, S3 Table, S3 Text). Likewise, lupin had the shortest shoots. The differences between aged and new residue were relatively small, although aged residue tended to have longer shoots, except for barley, indicating less herbicide availability in the residue. This was particularly in the case for wheat and chickpea, with and without rainfall, and for lupin with no rainfall. Rainfall generally increased shoot length and produced similar results to prosulfocarb, where it had the greatest effect on chickpea residue, a smaller effect on barley residue and little effect on aged lupin residue.

As for the other two herbicides, trifluralin generally had the greatest or equal shoot length with barley residue and smallest with lupin residue (Fig 6, S3 Table, S3 Text). With no rainfall, cucumber in aged chickpea residue had longer shoots than in new residue, and the reverse occurred with canola. Age had little effect on the other crop residue types. Rainfall generally increased shoot length, including aged lupin residue. Rainfall also had little effect on cucumber growth in canola residue.

In summary, herbicide remaining on the residue was influenced most by crop residue type. In general, barley residue had the greatest or equal cucumber shoot length (as a percentage of the equivalent untreated residue) followed by wheat, canola, chickpea and lupin residues. Although, the shoot length of bioassay species grown in wheat, canola and chickpea residue was variable and influenced slightly more by herbicide, crop residue age and rainfall. This indicates that the barley residue had the least bioavailable herbicide and lupin residue the most. The effect of residue age was smaller than residue type. Older residue tended to have greater shoot length than new residue, although the effect of age was minimal with barley and lupin residue and the reverse was true for canola residue, except for pyroxasulfone. Rainfall generally increased shoot length of cucumber, indicating that some herbicide was washed off the residue into the soil below. The rainfall effect was greatest with chickpea residue and least for barley residue. Rainfall did not affect aged lupin residue, except with trifluralin.

### Herbicide in the soil

**Fig 7:**
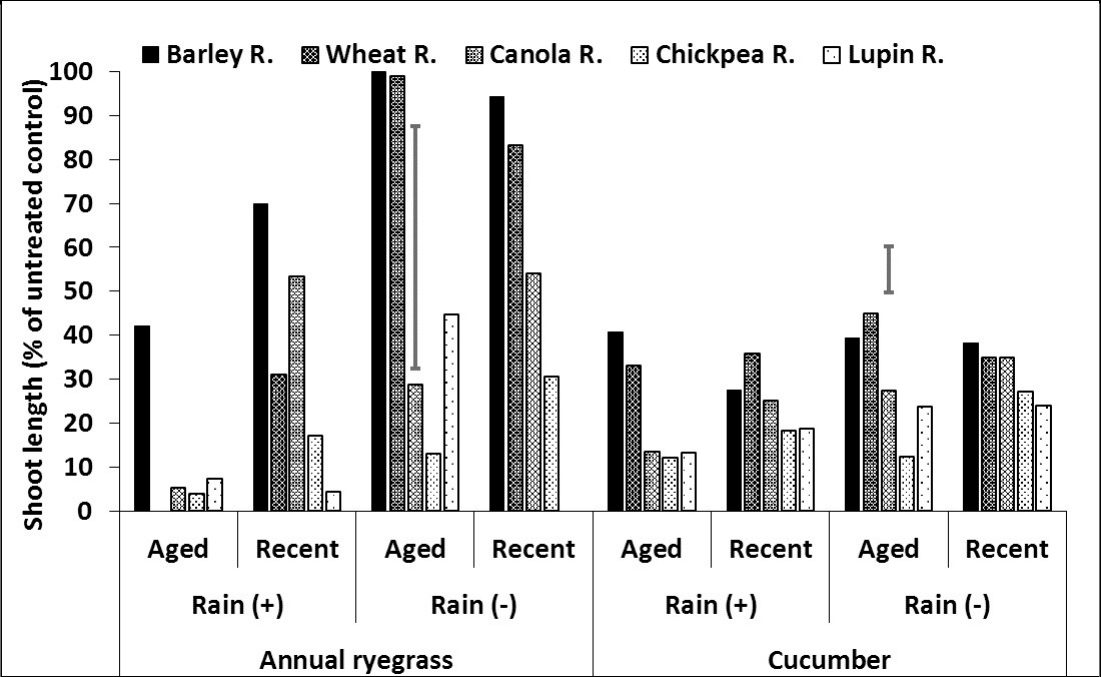
Effect of crop residue type and age on shoot length (% of untreated control) of ryegrass and cucumber in soil, after spraying with prosulfocarb followed by 20mm (+) or nil (–) rainfall. LSD bars show interactions between rainfall and age of residue at P = 0.05 for comparisons within the same bioassay species.

There were interactions between residue type, age and rainfall, with the responses greater for ryegrass than cucumber (Fig 7, S3 Table, S3 Text). For prosulfocarb in the absence of rainfall, barley and wheat residues had the greatest or equal shoot length, canola residue was intermediate and lupin and chickpea residues the least, although aged lupin residue had similar shoot length as aged canola residue (Fig 7, S3 Table, S3 Text). Aged chickpea and canola residue tended to have smaller shoot lengths in soil than new residue, although the differences were small, but significant. Rainfall generally reduced shoot length more with wheat than barley residue and this was particularly noticeable for aged wheat residue. Rainfall reduced shoot length to a smaller degree with the other residue types and ages, especially new canola residue.

In the situations with similar amounts of residue, prosulfocarb may wash into the soil least when applied to barley residue than the other residue types. While wheat residue had a similar effect to barley residue in the absence of rainfall, rainfall improved prosulfocarb efficacy more in wheat than barley residue. The effects of residue age on leaching of prosulfocarb into the soil were relatively small.

**Fig 8:**
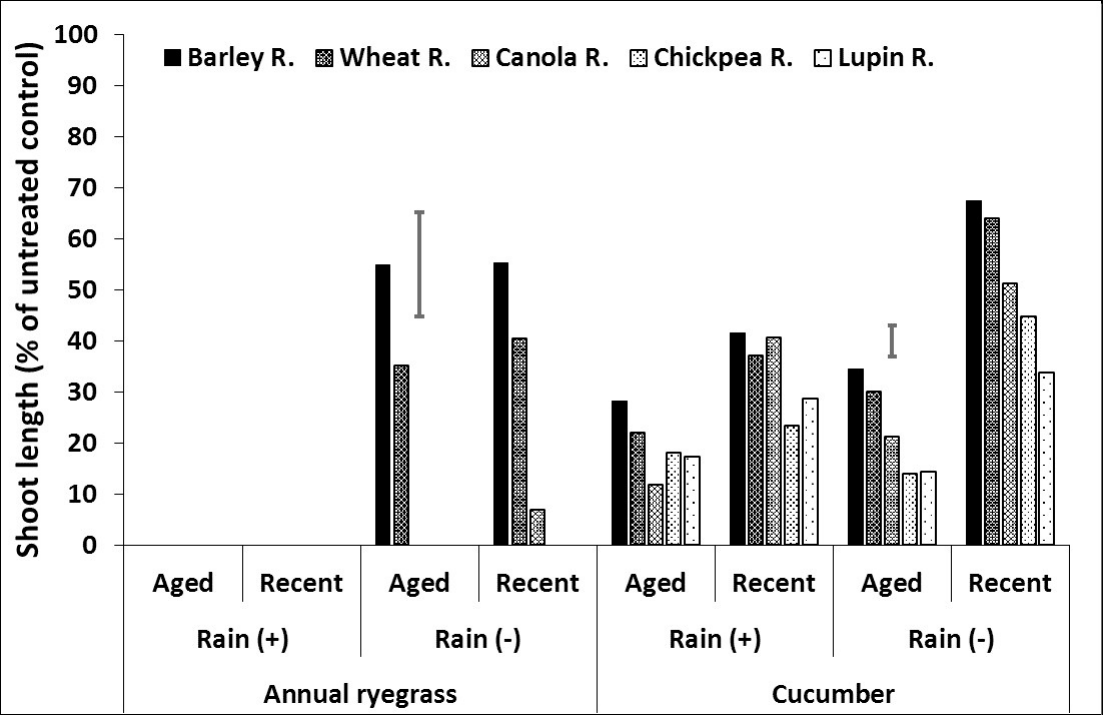
Effect of crop residue type and age on shoot length (% of untreated control) of ryegrass and cucumber in soil, after spraying with pyroxasulfone followed by 20mm (+) or nil (–) rainfall. LSD bars show interactions between rainfall and age of residue at P = 0.05 for comparisons within the same bioassay species.

For pyroxasulfone, the bioassay species in the soil generally had the greatest shoot length with barley and wheat residues, followed by canola, chickpea and lupin. The effect of age was relatively small and insignificant, but older residue consistently produced shorter shoot lengths in cucumber than new residue, suggesting that more herbicide reached the soil under older residue. Pyroxasulfone killed all the ryegrass in the soil after rainfall for all residue types and ages and, unlike prosulfocarb, rainfall effectiveness did not differ between barley and wheat (Fig 8, S3 Table, S3 Text). Rainfall also reduced shoot length in cucumber with both aged and new residue, although the effect was relatively small for aged residue.

**Fig 9:**
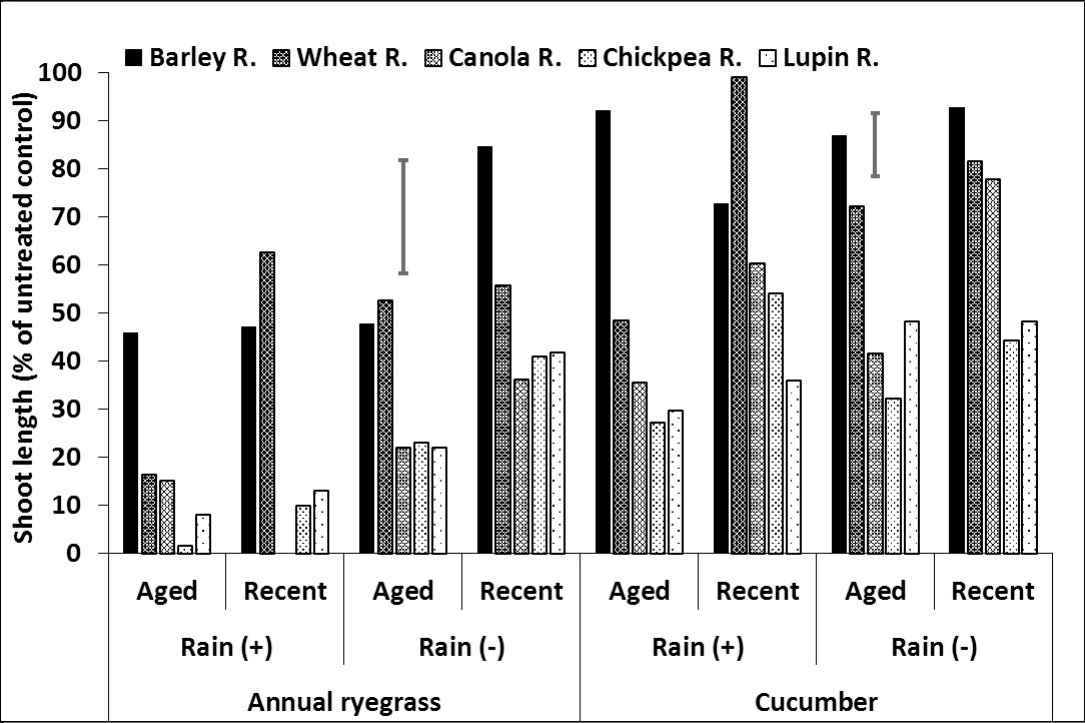
Effect of crop residue type and age on shoot length (% of untreated control) of ryegrass and cucumber in soil, after spraying with trifluralin followed by 20mm (+) or nil (–) rainfall. LSD bars show interactions between rainfall and age of residue at P = 0.05 for comparisons within the same bioassay species.

In the absence of rainfall, the effect of trifluralin and residue type on shoot length was similar to the other herbicides with the barley and wheat residues generally having the greatest and chickpea and lupin residues the shortest shoots; implying that more herbicide reached the soil under chickpea and lupin residues (Fig 9, S3 Table, S3 Text). With no rainfall, aged residue generally had more herbicide in the soil (shorter shoots) than new residue, but the effect was variable and relatively small for some residue types. Like the other herbicides, shoot length generally declined with rainfall, but the results were more variable for trifluralin; for example, rainfall did not affect shoot length with fresh wheat residue or aged barley residue (Fig 9, S3 Table, S3 Text).

**Fig 10:**
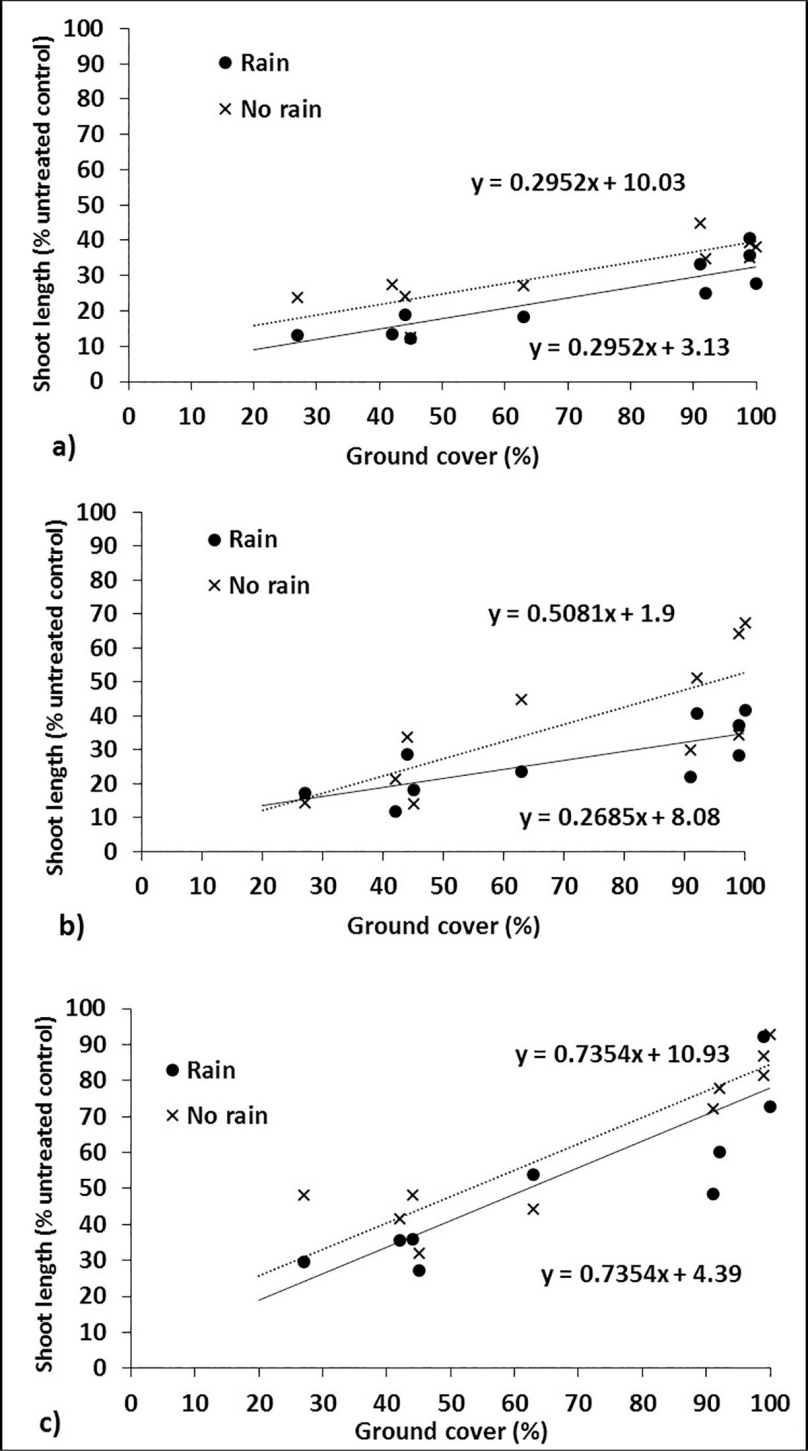
Relationship between shoot length (% untreated control) and ground cover (across the different residue types and ages) for a) prosulfocarb, b) pyroxasulfone and c) trifluralin.

Considering all three herbicides, shoot length in the soil was generally greatest or equal for barley residue followed by wheat residues, intermediate but variable for canola residue, and least for chickpea and lupin residues. This suggests that more herbicide reached the soil under chickpea and lupin residues and was mainly a function of ground cover. A linear regression of ground cover percentage against cucumber shoot length in the soil (% of untreated control) for all data (across all herbicides, residue types and ages) without simulated rainfall showed a significant, but weak, relationship with only 36% of the variance accounted for. However, this increased to 75% of the variance accounted for when the regression included herbicide as a factor i.e. separate linear regressions for each herbicide (Table 3). Further linear regression analysis, on individual herbicides, compared simulated rainfall with the nil rainfall control. As expected, the regression line for rainfall was lower than that of nil rainfall, because the plants were smaller due to more herbicide washing off the residue into the soil (Fig 10 and Table 3).

**Table 3:** Linear regressions of ground cover percentage against cucumber shoot length in the soil (% of untreated control) for a) all data with no simulated rainfall and herbicide (prosulfocarb, pyroxasulfone and trifluralin) as the grouping factor and b) prosulfocarb, c) pyroxasulfone and d) trifluralin comparing simulated rainfall (rain vs nil) as the grouping factor.

Regression description	Grouping factor	Intercept	Intercept P-value	Slope	Slope P-value	R^2^
**a) All data with nil rainfall**						
*No grouping factor (single line)*	Single line	9.2	0.048	0.49	<0.001	0.34
*Herbicide as grouping factor*	Prosulfocarb	11.6	0.020	0.27	<0.001	0.75
	Pyroxasulfone	1.9	0.700	0.51	<0.001	
	Trifluralin	14.2	0.005	0.69	<0.001	
**b) Prosulfocarb**						
*No grouping factor (single line)*	Single line	6.58	0.016	0.30	<0.001	0.46
*Rainfall as grouping factor*						
Compare nil rain slope and intercept vs reference of rain^†^	Nil rain	10.02	0.047	-0.04	0.500	0.54
Estimate of parallel lines (same slope different intercepts) ^†^	Rain	3.13	0.236	0.30	<0.001	0.54
	Nil rain	10.03	<0.001			
**c) Pyroxasulfone**						
*No grouping factor (single line)*	Single line	6.58	0.016	0.30	<0.001	0.46
*Rainfall as grouping factor*						
Compare nil rain slope and intercept vs reference of rain^‡^	Nil rain	-6.18	0.406	0.24	0.017	0.51
Estimate of separate lines (different slopes and intercepts) ^‡^	Rain	8.08	0.127	0.27	<0.001	0.51
	Nil rain	1.9	0.717	0.51	<0.001	
**d) Trifluralin**						
*No grouping factor (single line)*	Single line	7.66	0.088	0.74	<0.001	0.66
*Rainfall as grouping factor*						
Compare nil rain slope and intercept vs reference of rain*	Nil rain	12.99	0.140	-0.092	0.429	0.68
Estimate of parallel lines (same slope different intercepts) *	Rain	4.39	0.345	0.74	<0.001	0.68
	Nil rain	10.39	0.021			

^†^ Prosulfocarb–slopes not significantly different, therefore estimate of parallel lines given.

^‡^ Pyroxasulfone–slopes significantly different, therefore estimate of separate lines given.

* Trifluralin–slopes not significantly different, therefore estimate of parallel lines given.

Only pyroxasulfone had a significant interaction with rainfall (i.e. the slopes of the rain and nil rainfall lines differed significantly), with the slope of the regression decreasing from 0.51 in the absence of rainfall to 0.27 following rainfall (Table 3). This demonstrates the effectiveness of rainfall to reduce the impact of residue and improve the efficacy of pyroxasulfone, especially in the presence of thick crop residues (Fig 10). Overall, residue age only had a small effect on the amount of herbicide reaching the soil, with slightly more herbicide reaching the soil under aged than new residue.

In the absence of rainfall this would most likely be related to ground cover, with aged residue having lower ground cover percentage than new residue, with the differences more evident for canola, chickpea and lupin residues. The effect of lignin concentration (between residue types and ages) on the amount of herbicide washing off the residue was difficult to determine, as lignin was confounded with ground cover percentage. This analysis suggests that farmers simply need to assess residue ground cover, irrespective of its type, in order to assess the impact of residue on herbicide efficacy in the absence of rainfall or irrigation.

Rainfall after herbicide application improved the efficacy by leaching some intercepted herbicide into the soil beneath crop residues, which in turn better increased its availability and potential for weed control. In the absence of simulated rainfall, the less herbicide reached the soil with increasing residue amount, with a marked increase in shoot length from 2 to 4 t ha^–1^. Nonetheless, 100% ryegrass control was achieved with pyroxasulfone after rainfall, even at 4 t ha^–1^ residue. The results showed that, with rainfall or irrigation, pyroxasulfone can still be effective as a pre-emergent herbicide when the amount of flat residue on the soil surface exceeds 2 t ha^–1^ and prosulfocarb when the amount of flat residue on the soil surface is less than 2 t ha^–1^. In a situation with crop residue covering the soil, more prosulfocarb and trifluralin reached the soil after rainfall when the chemicals were sprayed onto initially dry compared with wet wheat residue. In contrast, the moisture status of the residue at the time of herbicide application had little effect on the amount of pyroxasulfone that reached the soil after subsequent rainfall. If possible, farmers should avoid spraying prosulfocarb and trifluralin into wet crop residue, otherwise they may need to increase the herbicide rate to compensate for losses when sprayed onto wet residues. Aslam et al. [58] showed that under a light and frequent rainfall regime, S-metolachlor dissipation in crop residues was quicker than a heavy and infrequent rain regime. This was due to wetter surface conditions, where crop residue decomposition was also faster. Microbial decomposition of crop residues is highly affected by water dynamics (rainfall) and temperature at the soil–residue interface [59–62]. Unger [20] reported that crop residues that are often wet have low sorption capacity for pesticides and are less aerodynamically stable than the soil underneath. Aged canola, chickpea and lupin residue generally had lower ground cover than new residue, while the differences were relatively small for aged and new cereals residues.

Crop residue type had a greater effect on herbicide interception than age of residue. With 4 t ha^–1^ of crop residue, the least herbicide reached the soil with barley residue followed by wheat with canola residue intermediate but variable, and chickpea and lupin residues the most. There was a strong positive linear relationship between ground cover percentage and growth of the bioassay species (i.e. less herbicide reached the soil with more ground cover). Lupin stubble had the least ground cover, followed by chickpea, canola and the cereals, which had > 90% ground cover at 4 t ha^–1^ of residue. The relative importance of percent ground cover compared with lignin concentration on herbicide leaching from residue is of interest, however, it could not be determined in this research as the two were confounded. Also, there was no consistent pattern between amount of herbicide leached and lignin concentration, possibly because of the relatively small differences in lignin concentration between residue types and ages.

## Supporting information

S1 Table Raw data from residue and soil used in the Figs 1 and 2 (Experiment1). (XLSX)

S2 Table Raw data from residue and soil used in the Fig 3 (Experiment2). (XLSX)

S3 Table Raw data from residue and soil used in the Figs 4–9 (Experiment3). (XLSX)

S1 Fig Original figure of Figs 1 and 2 cited in the text (Experiment1). (XLSX)

S2 Fig Original figure of Fig 3 cited in the text (Experiment2). (XLSX)

S3 Fig Original figures of Figs 4–9 cited in the text (Experiment3). (XLSX)

S1 Text Output of the analyses of data from Experiment1. (DOCX)

S2 Text Output of the analyses of data from Experiment2. (DOCX)

S3 Text Output of the analyses of data from Experiment3. (DOCX)

## References

[1] Verhulst N, Govaerts B, Verachtert E, Castellanos-Navarrete A, Mezzalama M, Wall P, et al Conservation agriculture, improving soil quality for sustainable production systems In: Lal R, Stewart BA, editors. Advances in Soil Science: Food Security and Soil Quality. FLorida, USA,: CRC Press, Boca Raton; 2010 p. 137–208.

[2] Kassam A, Friedrich T, Derpsch R, Lahmar R, Mrabet R, Basch G, et al Conservation agriculture in the dry Mediterranean climate. Field Crops Research. 2012;132:7–17.

[3] Serraj R, Siddique KH. Conservation agriculture in dry areas. Field Crops Research. 2012;132:1–6.

[4] Llewellyn RS, D’Emden FH, Kuehne G. Extensive use of no-tillage in grain growing regions of Australia. Field Crops Research. 2012;132:204–12.

[5] Unger PW, Wiese AF. Managing irrigated winter wheat residues for water storage and subsequent dryland grain sorghum production. Soil Science Society of America Journal. 1979;43(3):582–8.

[6] Prasad R, Power J. Crop residue management Advances in soil science: Springer; 1991 p. 205–51.

[7] Roper MM, Gupta V, Murphy DV. Tillage practices altered labile soil organic carbon and microbial function without affecting crop yields. Soil Research. 2010;48(3):274–85.

[8] Farooq M, Siddique KHM. Conservation Agriculture: Concepts, Brief History, and Impacts on Agricultural Systems In: Farooq M, Siddique KHM, editors. Conservation Agriculture: Springer, Cham; 2015.

[9] Banks PA, Robinson EL. The influence of straw mulch on the soil reception and persistence of metribuzin. Weed Science. 1982;3(3):164–8.

[10] Donald AC, Gail AW, Burnside OC. Effect of winter wheat (*Triticum aestivum*) straw mulch level on weed control. Weed Science. 1986;34(1):110–4.

[11] Ghadiri H, Shea PJ, Wicks GA. Interception and retention of atrazine by wheat (*Triticum aestivum* L.) stubble. Weed Science. 1984;32(1):24–7.

[12] Bauman TT, Ross MA. Effect of three tillage systems on the persistence of atrazine. Weed Science. 1983;31(3):423–6.

[13] Banks P, Robinson E, editors. Activity of acetochlor, alachlor, and metolachlor as affected by straw. Proceedings—Southern Weed Science Society; 1983.

[14] Strek H, Weber J. Alachlor (lasso) and metolachlor (dual) comparisons in conventional and reduced tillage systems [Weed control in corn]. Proceedings-Southern Weed Science Society (USA). 1981.

[15] Strek J, Weber J, editors. Adsorption, mobility, and activity comparisons between alachlor (Lasso) and metolachlor (Dual)[Barnyardgrass *Echinochloa crus-galli*, phytotoxicity, herbicides]. Proceedings Southern Weed Science Society; 1982.

[16] Prihar S, Sandhu K, Khera K. Maize (Zea mays L.) and weed growth, as affected by levels of straw mulching with and without herbicide under conventional and minimum tillage. Indian Journal of Ecology. 1975;2:13–22.

[17] Liebl RA, Worsham AD, editors. Tillage and mulch effects on morningglory (*Ipomoea spp*.) and certain other weed species. Proceedings of the Southern Weed Science Society; 1983.

[18] Crutchfield DA, Wicks GA, Burnside OC. Effect of winter wheat (*Triticum aestivum*) straw mulch level on weed control. Weed Science. 1986:110–4.

[19] Day BE. Principles of plant and animal pest control Day BE, editor. Washington, DC: National Academy of Sciences-National Research Council (Committee on, Plant Animal, Pests); 1968.

[20] Unger PW. Managing agricultural residues. Bushland, Texas, USA: Lewis Publishers; 1994. 448 p.

[21] Lal R. No-tillage effects on soil properties under different crops in Western Nigeria. Soil Science Society of America Journal. 1976;40(5):762–8.

[22] De Silva SHSA, Cook HF. Soil physical conditions and physiological performance of cowpea following organic matter amelioration of sandy substrates. Communications in Soil Science and Plant Analysis. 2003;34(7–8):1039–58.

[23] Banks PA, Robinson EL. Soil reception and activity of acetochlor, alachlor, and metolachlor as affected by wheat (*Triticum aestivum*) straw and irrigation. Weed Science. 1986;34(4):607–11.

[24] Morrison MW, Prunty L, Giles JF. Characterizing strength of soil crusts formed by simulated rainfall. Soil Science Society of America Journal. 1985;49(2):427–31.

[25] Dao TH. Field decay of wheat straw and its effects on metribuzin and S-ethyl metribuzin sorption and elution from crop residues. Journal of Environmental Quality. 1991;20(1):203–8.

[26] Grover R. Adsorption of picloram by soil colloids and various other adsorbents. Weed Science. 1971;19(4):417–8.

[27] Zablotowicz RM, Locke MA, Smeda RL. Degradation of 2, 4-D and fluometuron in cover crop residues. Chemosphere. 1998;37(1):87–101.

[28] Reddy KN, Locke MA, Wagner SC, Zablotowicz RM, Gaston LA, Smeda RJ. Chlorimuron ethyl sorption and desorption kinetics in soils and herbicide-desiccated cover crop residues. Journal of Agricultural and Food Chemistry. 1995;43(10):2752–7.

[29] Reddy KN, Locke MA, Gaston LA. Tillage and cover crop effects on cyanazine adsorption and desorption kinetics. Soil Science. 1997;162(7):501–9.

[30] Gaston L, Boquet D, Bosch M. Fluometuron wash-off from cover crop residues and fate in a loessial soil. Soil Science. 2001;166(10):681–90.

[31] Boutsalis P, Gill GS, Preston C. Incidence of herbicide resistance in rigid ryegrass (*Lolium rigidum*) across southeastern Australia. Weed Technology. 2012;26(3):391–8.

[32] Saini RK, Kleemann SGL, Preston C, Gill GS. Alternative herbicides for the management of clethodim-resistant rigid ryegrass (*Lolium rigidum*) in faba bean (*Vicia faba* L.) in Southern Australia. Weed Technology. 2015;29(3):578–86.

[33] Tanetani Y. Action mechanism of a novel herbicide, pyroxasulfone (Part 2)(Molecular target & mode of action, Poster, 1) Pest management, crop protection and vector control. Journal of Pesticide Science. 2011;36(1):152.

[34] Tanetani Y, Kaku K, Kawai K, Fujioka T, Shimizu T. Action mechanism of a novel herbicide, pyroxasulfone. Pesticide Biochemistry and Physiology. 2009;95(1):47–55.

[35] Tidemann BD. Potential for a Pyroxasulfone and Sulfentrazone Herbicide Combination to Control Herbicide-Resistant Weeds in Field Pea, as Affected by Edaphic Factors Edmonton, Alberta: University of Alberta; 2014.

[36] Mangin AR. Optimizing Pyroxasulfone Efficacy on Wild Oat (Avena fatua L.) [Master of Science thesis] Albert, Canada: University of Alberta; 2016.

[37] Bailly GC. Effectiveness of prosulfocarb-based treatments for the control of sensitive and herbicide resistant Lolium spp. populations. 2012.

[38] Busi R. Resistance to herbicides inhibiting the biosynthesis of very‐long‐chain fatty acids. Pest Management Science. 2014;70(9):1378–84. 10.1002/ps.3746 24482320 10.1002/ps.3746

[39] Busi R, Gaines TA, Walsh MJ, Powles SB. Understanding the potential for resistance evolution to the new herbicide pyroxasulfone: field selection at high doses versus recurrent selection at low doses. Weed Research. 2012;52(6):489–99.

[40] Busi R, Powles SB. Cross‐resistance to prosulfocarb+ S‐metolachlor and pyroxasulfone selected by either herbicide in *Lolium rigidum*. Pest Management Science. 2016;72(9):1664–72. 10.1002/ps.4253 26864800 10.1002/ps.4253

[41] Rayment GE, Lyons DJ. Soil Chemical Methods: Australasia: CSIRO Publishing; 2011.

[42] Boutsalis P, Gill GS, Preston C. Control of rigid ryegrass in Australian wheat production with pyroxasulfone. Weed Technology. 2014;28(2):332–9.

[43] Khalil Y, Siddique KHM, Ward P, Piggin C, Bong SH, Nambiar S, et al A bioassay for prosulfocarb, pyroxasulfone and trifluralin detection and quantification in soil and crop residues. Crop & Pasture Science. 2018;69:606–10.

[44] Dairy-One-Forage-Laboratory. Analytical procedures 2052 O’Neil Road, Macedon, NY 14502: ANKOM Technology; 2015 [updated July 2015; cited 2018 7/02/2018]. Available from: http://dairyone.com/wp-content/uploads/2014/02/Forage-Lab-Analytical-Procedures-Listing-Alphabetical-July-2015.pdf.

[45] Lamari L. ASSESS 2.0: Image Analysis Software for Plant Disease Quantification Department of Plant Science, University of Manitoba, Winnipeg, R3T2N2, Canada The American Phytopathological Society (APS); 2008.

[46] Meyer LD. Use of the rainulator for runoff plot research. Soil Science Society of America Journal. 1960;24(4):319–22.

[47] Hermsmeier LF, Meyer L, Barnett A, Young R. Construction and operation of a 16-unit rainulator Washington DC, USA: Agricultural Research Service, U.S. Department of Agriculture; 1963.

[48] Somasegaran P, Hoben HJ. Methods in legume-Rhizobium technology: University of Hawaii NifTAL Project and MIRCEN, Department of Agronomy and Soil Science, Hawaii Institute of Tropical Agriculture and Human Resources, College of Tropical Agriculture and Human Resources; 1985.

[49] Payne RW, Murray DA, Harding SA, Baird DB, Soutar DM. GenStat for Windows (12th Edition) Introduction VSN International, Hemel Hempstead 2009.

[50] Erbach DC, Lovely WG. Effect of plant residue on herbicide performance in no-tillage corn. Weed Science. 1975:512–5.

[51] Chauhan BS, Gill GS, Preston C. Tillage system effects on weed ecology, herbicide activity and persistence: a review. Animal Production Science. 2006;46(12):1557–70.

[52] Borger CPD, Riethmuller GP, Ashworth M, Minkey D, Hashem A, Powles SB. Increased carrier volume improves preemergence control of rigid ryegrass (*Lolium rigidum*) in zero-tillage seeding systems. Weed Technology. 2013;27(4):649–55.

[53] Reinertsen SA, Elliott LF, Cochran VL, Campbell GS. Role of available carbon and nitrogen in determining the rate of wheat straw decomposition. Soil Biology and Biochemistry. 1984;16(5):459–64.

[54] Leonard L. Managing for stubble retention Department of Agriculture and Food, Western Australia, Perth: 1993.

[55] Leys JF, Heinjus D. Simulated wind erosion in the South Australian Murray Mallee: Conservation Service of NSW; 1991.

[56] Martin CD, Baker JL, Erbach DC, Johnson HP. Washoff of Herbicides Applied to Corn Residue. Transactions of the ASAE. 1978;21(6):1164.

[57] Dang A, Silburn M, Craig I, Shaw M, Foley J. Washoff of residual photosystem II Herbicides from sugar cane trash under a rainfall simulator. Journal of Agricultural and Food Chemistry. 2016;64(20):3967–74. 10.1021/acs.jafc.5b04717 26964670 10.1021/acs.jafc.5b04717

[58] Aslam S, Iqbal A, Deschamps M, Recous S, Garnier P, Benoit P. Effect of rainfall regimes and mulch decomposition on the dissipation and leaching of S-metolachlor and glyphosate: a soil column experiment. Pest Management Science. 2015;71(2):278–91. 10.1002/ps.3803 24753267 10.1002/ps.3803

[59] Coppens F, Garnier P, De Gryze S, Merckx R, Recous S. Soil moisture, carbon and nitrogen dynamics following incorporation and surface application of labelled crop residues in soil columns. European Journal of Soil Science. 2006;57(6):894–905.

[60] Taylor-Lovell S, Sims GK, Wax LM. Effects of moisture, temperature, and biological activity on the degradation of isoxaflutole in soil. Journal of Agricultural and Food Chemistry. 2002;50(20):5626–33. 12236689 10.1021/jf011486l

[61] Isensee AR, Sadeghi AM. Long-term effect of tillage and rainfall on herbicide leaching to shallow groundwater. Chemosphere. 1995;30(4):671–85.

[62] Issa S, Wood M. Degradation of atrazine and isoproturon in surface and sub‐surface soil materials undergoing different moisture and aeration conditions. Pest Management Science. 2005;61(2):126–32. 10.1002/ps.951 15619717 10.1002/ps.951

