# Supplementary material for: Effect of crop residues on interception and activity of prosulfocarb, pyroxasulfone, and trifluralin
Source: PLoS One. 2018 Dec 6;13(12):e0208274. doi: 10.1371/journal.pone.0208274 (PMC6283640; doi:10.1371/journal.pone.0208274)
Supplement: S1 Text — (DOCX) [file pone.0208274.s007.docx]

**Each herbicide with each plant species considered one experiment**:

220 "General Analysis of Variance."
 221 BLOCK Rep/PlotNo
 222 TREATMENTS Trmt*Res_Amo
 223 COVARIATE "No Covariate"
 224 ANOVA [PRINT=aovtable,information,means,%cv; FACT=32; CONTRASTS=7; PCONTRASTS=7; FPROB=yes;\
 225 PSE=diff,lsd; LSDLEVEL=5] SL%_of_Ctrl

Analysis of variance (Pro-Res-AR)

Variate: SL%_of_Ctrl

Source of variation d.f. s.s. m.s. v.r. F pr.

Rep stratum 3 0. 0.

Rep.PlotNo stratum

Trmt 1 0. 0.

Res_Amo 3 0. 0.

Trmt.Res_Amo 3 0. 0.

Residual 21 0. 0.

Total 31 0.

Tables of means

Variate: SL%_of_Ctrl

Grand mean 0.00

Trmt (+)Rain (-)Rain UTC

0.00 0.00

Res_Amo 0 1 2 4

0.00 0.00 0.00 0.00

Res_Amo Ctrl

Trmt Res_Amo 0 1 2

(+)Rain 0.00 0.00 0.00

(-)Rain 0.00 0.00 0.00

Trmt Res_Amo 4 Ctrl

(+)Rain 0.00

(-)Rain 0.00

Standard errors of differences of means

Table Trmt Res_Amo Trmt

Res_Amo

rep. 16 8 4

d.f. * * *

s.e.d. 0.000 0.000 0.000

Least significant differences of means (5% level)

Table Trmt Res_Amo Trmt

Res_Amo

rep. 16 8 4

d.f. * * *

l.s.d. * * *

Stratum standard errors and coefficients of variation

Variate: SL%_of_Ctrl

Stratum d.f. s.e. cv%

Rep 3 0.000 0.0

Rep.PlotNo 21 0.000 0.0

Analysis of variance (Pro-Soil-AR)

Variate: SL%_of_Ctrl

Source of variation d.f. s.s. m.s. v.r. F pr.

Rep stratum 3 314.3 104.8 0.90

Rep.PlotNo stratum

Trmt 1 1118.6 1118.6 9.57 0.006

Res_Amo 3 16474.1 5491.4 46.96 <.001

Trmt.Res_Amo 3 974.4 324.8 2.78 0.066

Residual 21 2455.9 116.9

Total 31 21337.3

Tables of means

Variate: SL%_of_Ctrl

Grand mean 19.82

Trmt (+)Rain (-)Rain UTC

13.91 25.74

Res_Amo 0 1 2 4

0.00 6.02 15.30 57.97

Res_Amo Ctrl

Trmt Res_Amo 0 1 2

(+)Rain 0.00 0.00 12.40

(-)Rain 0.00 12.03 18.21

Trmt Res_Amo 4 Ctrl

(+)Rain 43.24

(-)Rain 72.70

Standard errors of differences of means

Table Trmt Res_Amo Trmt

Res_Amo

rep. 16 8 4

d.f. 21 21 21

s.e.d. 3.823 5.407 7.647

Least significant differences of means (5% level)

Table Trmt Res_Amo Trmt

Res_Amo

rep. 16 8 4

d.f. 21 21 21

l.s.d. 7.951 11.245 15.902

Stratum standard errors and coefficients of variation

Variate: SL%_of_Ctrl

Stratum d.f. s.e. cv%

Rep 3 3.619 18.3

Rep.PlotNo 21 10.814 54.6

Analysis of variance (Pro-Res-CU)

Variate: SL%_of_Ctrl

Source of variation d.f. s.s. m.s. v.r. F pr.

Rep stratum 3 295.56 98.52 5.61

Rep.PlotNo stratum

Trmt 1 1321.39 1321.39 75.22 <.001

Res_Amo 3 18859.97 6286.66 357.85 <.001

Trmt.Res_Amo 3 584.79 194.93 11.10 <.001

Residual 21 368.92 17.57

Total 31 21430.64

Tables of means

Variate: SL%_of_Ctrl

Grand mean 41.06

Trmt (+)Rain (-)Rain UTC

47.49 34.63

Res_Amo 0 1 2 4

0.00 47.51 54.43 62.30

Res_Amo Ctrl

Trmt Res_Amo 0 1 2

(+)Rain 0.00 54.00 61.62

(-)Rain 0.00 41.01 47.23

Trmt Res_Amo 4 Ctrl

(+)Rain 74.32

(-)Rain 50.29

Standard errors of differences of means

Table Trmt Res_Amo Trmt

Res_Amo

rep. 16 8 4

d.f. 21 21 21

s.e.d. 1.482 2.096 2.964

Least significant differences of means (5% level)

Table Trmt Res_Amo Trmt

Res_Amo

rep. 16 8 4

d.f. 21 21 21

l.s.d. 3.082 4.358 6.163

Stratum standard errors and coefficients of variation

Variate: SL%_of_Ctrl

Stratum d.f. s.e. cv%

Rep 3 3.509 8.5

Rep.PlotNo 21 4.191 10.2

Analysis of variance (Pro-Soil-CU)

Variate: SL%_of_Ctrl

Source of variation d.f. s.s. m.s. v.r. F pr.

Rep stratum 3 129.0 43.0 0.38

Rep.PlotNo stratum

Trmt 1 0.8 0.8 0.01 0.932

Res_Amo 3 11732.7 3910.9 34.74 <.001

Trmt.Res_Amo 3 460.9 153.6 1.36 0.281

Residual 21 2364.4 112.6

Total 31 14687.8

Tables of means

Variate: SL%_of_Ctrl

Grand mean 60.85

Trmt (+)Rain (-)Rain UTC

61.02 60.69

Res_Amo 0 1 2 4

38.91 49.97 64.47 90.06

Res_Amo Ctrl

Trmt Res_Amo 0 1 2

(+)Rain 40.70 52.31 58.10

(-)Rain 37.11 47.63 70.85

Trmt Res_Amo 4 Ctrl

(+)Rain 92.96

(-)Rain 87.17

Standard errors of differences of means

Table Trmt Res_Amo Trmt

Res_Amo

rep. 16 8 4

d.f. 21 21 21

s.e.d. 3.752 5.305 7.503

Least significant differences of means (5% level)

Table Trmt Res_Amo Trmt

Res_Amo

rep. 16 8 4

d.f. 21 21 21

l.s.d. 7.802 11.033 15.603

Stratum standard errors and coefficients of variation

Variate: SL%_of_Ctrl

Stratum d.f. s.e. cv%

Rep 3 2.318 3.8

Rep.PlotNo 21 10.611 17.4

Analysis of variance (Pyro-Res-AR)

Variate: SL%_of_Ctrl

Source of variation d.f. s.s. m.s. v.r. F pr.

Rep stratum 3 0. 0.

Rep.PlotNo stratum

Trmt 1 0. 0.

Res_Amo 3 0. 0.

Trmt.Res_Amo 3 0. 0.

Residual 21 0. 0.

Total 31 0.

Tables of means

Variate: SL%_of_Ctrl

Grand mean 0.00

Trmt (+)Rain (-)Rain UTC

0.00 0.00

Res_Amo 0 1 2 4

0.00 0.00 0.00 0.00

Res_Amo Ctrl

Trmt Res_Amo 0 1 2

(+)Rain 0.00 0.00 0.00

(-)Rain 0.00 0.00 0.00

Trmt Res_Amo 4 Ctrl

(+)Rain 0.00

(-)Rain 0.00

Standard errors of differences of means

Table Trmt Res_Amo Trmt

Res_Amo

rep. 16 8 4

d.f. * * *

s.e.d. 0.000 0.000 0.000

Least significant differences of means (5% level)

Table Trmt Res_Amo Trmt

Res_Amo

rep. 16 8 4

d.f. * * *

l.s.d. * * *

Stratum standard errors and coefficients of variation

Variate: SL%_of_Ctrl

Stratum d.f. s.e. cv%

Rep 3 0.000 0.0

Rep.PlotNo 21 0.000 0.0

Analysis of variance (Pyro-Soil-AR)

Variate: SL%_of_Ctrl

Source of variation d.f. s.s. m.s. v.r. F pr.

Rep stratum 3 153.2 51.1 0.36

Rep.PlotNo stratum

Trmt 1 3662.1 3662.1 26.01 <.001

Res_Amo 3 4949.4 1649.8 11.72 <.001

Trmt.Res_Amo 3 4949.4 1649.8 11.72 <.001

Residual 21 2956.5 140.8

Total 31 16670.7

Tables of means

Variate: SL%_of_Ctrl

Grand mean 10.70

Trmt (+)Rain (-)Rain UTC

0.00 21.40

Res_Amo 0 1 2 4

0.00 0.00 12.43 30.37

Res_Amo Ctrl

Trmt Res_Amo 0 1 2

(+)Rain 0.00 0.00 0.00

(-)Rain 0.00 0.00 24.85

Trmt Res_Amo 4 Ctrl

(+)Rain 0.00

(-)Rain 60.73

Standard errors of differences of means

Table Trmt Res_Amo Trmt

Res_Amo

rep. 16 8 4

d.f. 21 21 21

s.e.d. 4.195 5.933 8.390

Least significant differences of means (5% level)

Table Trmt Res_Amo Trmt

Res_Amo

rep. 16 8 4

d.f. 21 21 21

l.s.d. 8.724 12.338 17.448

Stratum standard errors and coefficients of variation

Variate: SL%_of_Ctrl

Stratum d.f. s.e. cv%

Rep 3 2.527 23.6

Rep.PlotNo 21 11.865 110.9

Analysis of variance (Pyro-Res-CU)

Variate: SL%_of_Ctrl

Source of variation d.f. s.s. m.s. v.r. F pr.

Rep stratum 3 352.86 117.62 3.73

Rep.PlotNo stratum

Trmt 1 5461.15 5461.15 173.04 <.001

Res_Amo 3 17228.82 5742.94 181.97 <.001

Trmt.Res_Amo 3 2228.33 742.78 23.54 <.001

Residual 21 662.76 31.56

Total 31 25933.93

Tables of means

Variate: SL%_of_Ctrl

Grand mean 33.32

Trmt (+)Rain (-)Rain UTC

46.39 20.26

Res_Amo 0 1 2 4

0.00 23.25 54.83 55.21

Res_Amo Ctrl

Trmt Res_Amo 0 1 2

(+)Rain 0.00 46.49 69.22

(-)Rain 0.00 0.00 40.45

Trmt Res_Amo 4 Ctrl

(+)Rain 69.83

(-)Rain 40.59

Standard errors of differences of means

Table Trmt Res_Amo Trmt

Res_Amo

rep. 16 8 4

d.f. 21 21 21

s.e.d. 1.986 2.809 3.972

Least significant differences of means (5% level)

Table Trmt Res_Amo Trmt

Res_Amo

rep. 16 8 4

d.f. 21 21 21

l.s.d. 4.131 5.841 8.261

Stratum standard errors and coefficients of variation

Variate: SL%_of_Ctrl

Stratum d.f. s.e. cv%

Rep 3 3.834 11.5

Rep.PlotNo 21 5.618 16.9

Analysis of variance (Pyro-Soil-CU)

Variate: SL%_of_Ctrl

Source of variation d.f. s.s. m.s. v.r. F pr.

Rep stratum 3 549.30 183.10 13.37

Rep.PlotNo stratum

Trmt 1 1250.76 1250.76 91.35 <.001

Res_Amo 3 5495.77 1831.92 133.79 <.001

Trmt.Res_Amo 3 454.98 151.66 11.08 <.001

Residual 21 287.54 13.69

Total 31 8038.35

Tables of means

Variate: SL%_of_Ctrl

Grand mean 45.80

Trmt (+)Rain (-)Rain UTC

39.55 52.05

Res_Amo 0 1 2 4

27.85 41.70 49.56 64.08

Res_Amo Ctrl

Trmt Res_Amo 0 1 2

(+)Rain 23.38 39.52 43.54

(-)Rain 32.33 43.88 55.57

Trmt Res_Amo 4 Ctrl

(+)Rain 51.74

(-)Rain 76.42

Standard errors of differences of means

Table Trmt Res_Amo Trmt

Res_Amo

rep. 16 8 4

d.f. 21 21 21

s.e.d. 1.308 1.850 2.617

Least significant differences of means (5% level)

Table Trmt Res_Amo Trmt

Res_Amo

rep. 16 8 4

d.f. 21 21 21

l.s.d. 2.721 3.848 5.441

Stratum standard errors and coefficients of variation

Variate: SL%_of_Ctrl

Stratum d.f. s.e. cv%

Rep 3 4.784 10.4

Rep.PlotNo 21 3.700 8.1

Analysis of variance (Tri-Res-AR)

Variate: SL%_of_Ctrl

Source of variation d.f. s.s. m.s. v.r. F pr.

Rep stratum 3 182.86 60.95 1.73

Rep.PlotNo stratum

Trmt 1 170.75 170.75 4.84 0.039

Res_Amo 3 103.26 34.42 0.98 0.423

Trmt.Res_Amo 3 103.26 34.42 0.98 0.423

Residual 21 740.47 35.26

Total 31 1300.60

Tables of means

Variate: SL%_of_Ctrl

Grand mean 2.31

Trmt (+)Rain (-)Rain UTC

4.62 0.00

Res_Amo 0 1 2 4

0.00 5.05 2.08 2.11

Res_Amo Ctrl

Trmt Res_Amo 0 1 2

(+)Rain 0.00 10.09 4.17

(-)Rain 0.00 0.00 0.00

Trmt Res_Amo 4 Ctrl

(+)Rain 4.22

(-)Rain 0.00

Standard errors of differences of means

Table Trmt Res_Amo Trmt

Res_Amo

rep. 16 8 4

d.f. 21 21 21

s.e.d. 2.099 2.969 4.199

Least significant differences of means (5% level)

Table Trmt Res_Amo Trmt

Res_Amo

rep. 16 8 4

d.f. 21 21 21

l.s.d. 4.366 6.174 8.732

Stratum standard errors and coefficients of variation

Variate: SL%_of_Ctrl

Stratum d.f. s.e. cv%

Rep 3 2.760 119.5

Rep.PlotNo 21 5.938 257.1

Analysis of variance (Tri-Soil-AR)

Variate: SL%_of_Ctrl

Source of variation d.f. s.s. m.s. v.r. F pr.

Rep stratum 3 604.4 201.5 1.00

Rep.PlotNo stratum

Trmt 1 506.3 506.3 2.52 0.127

Res_Amo 3 33382.8 11127.6 55.44 <.001

Trmt.Res_Amo 3 3886.4 1295.5 6.45 0.003

Residual 21 4215.1 200.7

Total 31 42594.9

Tables of means

Variate: SL%_of_Ctrl

Grand mean 30.82

Trmt (+)Rain (-)Rain UTC

26.84 34.80

Res_Amo 0 1 2 4

0.00 12.32 26.63 84.33

Res_Amo Ctrl

Trmt Res_Amo 0 1 2

(+)Rain 0.00 21.00 23.59

(-)Rain 0.00 3.64 29.67

Trmt Res_Amo 4 Ctrl

(+)Rain 62.78

(-)Rain 105.88

Standard errors of differences of means

Table Trmt Res_Amo Trmt

Res_Amo

rep. 16 8 4

d.f. 21 21 21

s.e.d. 5.009 7.084 10.018

Least significant differences of means (5% level)

Table Trmt Res_Amo Trmt

Res_Amo

rep. 16 8 4

d.f. 21 21 21

l.s.d. 10.417 14.731 20.833

Stratum standard errors and coefficients of variation

Variate: SL%_of_Ctrl

Stratum d.f. s.e. cv%

Rep 3 5.018 16.3

Rep.PlotNo 21 14.167 46.0

Analysis of variance (Tri-Res-CU)

Variate: SL%_of_Ctrl

Source of variation d.f. s.s. m.s. v.r. F pr.

Rep stratum 3 430.30 143.43 5.93

Rep.PlotNo stratum

Trmt 1 1559.82 1559.82 64.44 <.001

Res_Amo 3 19921.97 6640.66 274.33 <.001

Trmt.Res_Amo 3 891.62 297.21 12.28 <.001

Residual 21 508.35 24.21

Total 31 23312.06

Tables of means

Variate: SL%_of_Ctrl

Grand mean 42.85

Trmt (+)Rain (-)Rain UTC

49.83 35.87

Res_Amo 0 1 2 4

0.00 53.87 55.16 62.37

Res_Amo Ctrl

Trmt Res_Amo 0 1 2

(+)Rain 0.00 58.78 63.72

(-)Rain 0.00 48.96 46.60

Trmt Res_Amo 4 Ctrl

(+)Rain 76.83

(-)Rain 47.91

Standard errors of differences of means

Table Trmt Res_Amo Trmt

Res_Amo

rep. 16 8 4

d.f. 21 21 21

s.e.d. 1.740 2.460 3.479

Least significant differences of means (5% level)

Table Trmt Res_Amo Trmt

Res_Amo

rep. 16 8 4

d.f. 21 21 21

l.s.d. 3.618 5.116 7.235

Stratum standard errors and coefficients of variation

Variate: SL%_of_Ctrl

Stratum d.f. s.e. cv%

Rep 3 4.234 9.9

Rep.PlotNo 21 4.920 11.5

Analysis of variance (Tri-Soil-CU)

Variate: SL%_of_Ctrl

Source of variation d.f. s.s. m.s. v.r. F pr.

Rep stratum 3 500.21 166.74 4.91

Rep.PlotNo stratum

Trmt 1 118.52 118.52 3.49 0.076

Res_Amo 3 7799.24 2599.75 76.59 <.001

Trmt.Res_Amo 3 344.75 114.92 3.39 0.037

Residual 21 712.82 33.94

Total 31 9475.55

Tables of means

Variate: SL%_of_Ctrl

Grand mean 71.03

Trmt (+)Rain (-)Rain UTC

72.95 69.10

Res_Amo 0 1 2 4

49.11 68.36 73.69 92.95

Res_Amo Ctrl

Trmt Res_Amo 0 1 2

(+)Rain 50.18 72.10 70.74

(-)Rain 48.04 64.62 76.65

Trmt Res_Amo 4 Ctrl

(+)Rain 98.78

(-)Rain 87.11

Standard errors of differences of means

Table Trmt Res_Amo Trmt

Res_Amo

rep. 16 8 4

d.f. 21 21 21

s.e.d. 2.060 2.913 4.120

Least significant differences of means (5% level)

Table Trmt Res_Amo Trmt

Res_Amo

rep. 16 8 4

d.f. 21 21 21

l.s.d. 4.284 6.058 8.567

Stratum standard errors and coefficients of variation

Variate: SL%_of_Ctrl

Stratum d.f. s.e. cv%

Rep 3 4.565 6.4

Rep.PlotNo 21 5.826 8.2
