# Supplementary material for: Effect of crop residues on interception and activity of prosulfocarb, pyroxasulfone, and trifluralin
Source: PLoS One. 2018 Dec 6;13(12):e0208274. doi: 10.1371/journal.pone.0208274 (PMC6283640; doi:10.1371/journal.pone.0208274)
Supplement: S2 Text — (DOCX) [file pone.0208274.s008.docx]

**Each herbicide with each plant species considered one experiment**:

190 "General Analysis of Variance."
 191 BLOCK Rep/PlotNo
 192 TREATMENTS Res_Con
 193 COVARIATE "No Covariate"

Analysis of variance (Pro-Res- AR)

Variate: SL%_of_Ctrl

Source of variation d.f. s.s. m.s. v.r. F pr.

Rep stratum 3 0.00 0.00

Rep.PlotNo stratum

Res_Con 3 30000.00 10000.00

Residual 9 0.00 0.00

Total 15 30000.00

Tables of means

Variate: SL%_of_Ctrl

Grand mean 25.00

Res_Con Dry TC

0.00 0.00

Res_Con UTC Wet

100.00 0.00

Standard errors of differences of means

Table Res_Con

rep. 4

d.f. *

s.e.d. 0.000

Least significant differences of means (5% level)

Table Res_Con

rep. 4

d.f. *

l.s.d. *

Stratum standard errors and coefficients of variation

Variate: SL%_of_Ctrl

Stratum d.f. s.e. cv%

Rep 3 0.000 0.0

Rep.PlotNo 9 0.000 0.0

Analysis of variance (Pro-Res-CU)

Variate: SL%_of_Ctrl

Source of variation d.f. s.s. m.s. v.r. F pr.

Rep stratum 3 91.77 30.59 0.66

Rep.PlotNo stratum

Res_Con 3 6934.56 2311.52 49.96 <.001

Residual 9 416.41 46.27

Total 15 7442.73

Tables of means

Variate: SL%_of_Ctrl

Grand mean 68.6

Res_Con Dry TC

70.8 42.3

Res_Con UTC Wet

100.0 61.4

Standard errors of differences of means

Table Res_Con

rep. 4

d.f. 9

s.e.d. 4.81

Least significant differences of means (5% level)

Table Res_Con

rep. 4

d.f. 9

l.s.d. 10.88

Stratum standard errors and coefficients of variation

Variate: SL%_of_Ctrl

Stratum d.f. s.e. cv%

Rep 3 2.77 4.0

Rep.PlotNo 9 6.80 9.9

Analysis of variance (Pro-Soil- AR)

Variate: SL%_of_Ctrl

Source of variation d.f. s.s. m.s. v.r. F pr.

Rep stratum 3 435.86 145.29 1.50

Rep.PlotNo stratum

Res_Con 3 15269.59 5089.86 52.57 <.001

Residual 9 871.37 96.82

Total 15 16576.82

Tables of means

Variate: SL%_of_Ctrl

Grand mean 55.87

Res_Con Dry TC

14.62 63.50

Res_Con UTC Wet

100.00 45.37

Standard errors of differences of means

Table Res_Con

rep. 4

d.f. 9

s.e.d. 6.958

Least significant differences of means (5% level)

Table Res_Con

rep. 4

d.f. 9

l.s.d. 15.739

Stratum standard errors and coefficients of variation

Variate: SL%_of_Ctrl

Stratum d.f. s.e. cv%

Rep 3 6.027 10.8

Rep.PlotNo 9 9.840 17.6

Analysis of variance (Pro-Soil-CU)

Variate: SL%_of_Ctrl

Source of variation d.f. s.s. m.s. v.r. F pr.

Rep stratum 3 83.50 27.83 0.59

Rep.PlotNo stratum

Res_Con 3 3269.35 1089.78 23.10 <.001

Residual 9 424.51 47.17

Total 15 3777.35

Tables of means

Variate: SL%_of_Ctrl

Grand mean 79.64

Res_Con Dry TC

70.55 85.33

Res_Con UTC Wet

100.00 62.67

Standard errors of differences of means

Table Res_Con

rep. 4

d.f. 9

s.e.d. 4.856

Least significant differences of means (5% level)

Table Res_Con

rep. 4

d.f. 9

l.s.d. 10.986

Stratum standard errors and coefficients of variation

Variate: SL%_of_Ctrl

Stratum d.f. s.e. cv%

Rep 3 2.638 3.3

Rep.PlotNo 9 6.868 8.6

Analysis of variance (Pyro-Res-AR)

Variate: SL%_of_Ctrl

Source of variation d.f. s.s. m.s. v.r. F pr.

Rep stratum 3 0.00 0.00

Rep.PlotNo stratum

Res_Con 3 30000.00 10000.00

Residual 9 0.00 0.00

Total 15 30000.00

Tables of means

Variate: SL%_of_Ctrl

Grand mean 25.00

Res_Con Dry TC

0.00 0.00

Res_Con UTC Wet

100.00 0.00

Standard errors of differences of means

Table Res_Con

rep. 4

d.f. *

s.e.d. 0.000

Least significant differences of means (5% level)

Table Res_Con

rep. 4

d.f. *

l.s.d. *

Stratum standard errors and coefficients of variation

Variate: SL%_of_Ctrl

Stratum d.f. s.e. cv%

Rep 3 0.000 0.0

Rep.PlotNo 9 0.000 0.0

Analysis of variance (Pyro-Res-CU)

Variate: SL%_of_Ctrl

Source of variation d.f. s.s. m.s. v.r. F pr.

Rep stratum 3 228.85 76.28 1.21

Rep.PlotNo stratum

Res_Con 3 13763.19 4587.73 72.70 <.001

Residual 9 567.93 63.10

Total 15 14559.98

Tables of means

Variate: SL%_of_Ctrl

Grand mean 56.8

Res_Con Dry TC

56.7 17.3

Res_Con UTC Wet

100.0 53.3

Standard errors of differences of means

Table Res_Con

rep. 4

d.f. 9

s.e.d. 5.62

Least significant differences of means (5% level)

Table Res_Con

rep. 4

d.f. 9

l.s.d. 12.71

Stratum standard errors and coefficients of variation

Variate: SL%_of_Ctrl

Stratum d.f. s.e. cv%

Rep 3 4.37 7.7

Rep.PlotNo 9 7.94 14.0

Analysis of variance (Pyro-Soil-AR)

Variate: SL%_of_Ctrl

Source of variation d.f. s.s. m.s. v.r. F pr.

Rep stratum 3 167.82 55.94 1.00

Rep.PlotNo stratum

Res_Con 3 27206.05 9068.68 162.11 <.001

Residual 9 503.47 55.94

Total 15 27877.33

Tables of means

Variate: SL%_of_Ctrl

Grand mean 36.69

Res_Con Dry TC

0.00 46.74

Res_Con UTC Wet

100.00 0.00

Standard errors of differences of means

Table Res_Con

rep. 4

d.f. 9

s.e.d. 5.289

Least significant differences of means (5% level)

Table Res_Con

rep. 4

d.f. 9

l.s.d. 11.964

Stratum standard errors and coefficients of variation

Variate: SL%_of_Ctrl

Stratum d.f. s.e. cv%

Rep 3 3.740 10.2

Rep.PlotNo 9 7.479 20.4

Analysis of variance (Pyro-Soil-CU)

Variate: SL%_of_Ctrl

Source of variation d.f. s.s. m.s. v.r. F pr.

Rep stratum 3 15.39 5.13 0.09

Rep.PlotNo stratum

Res_Con 3 10440.01 3480.00 59.97 <.001

Residual 9 522.22 58.02

Total 15 10977.62

Tables of means

Variate: SL%_of_Ctrl

Grand mean 65.51

Res_Con Dry TC

38.65 80.00

Res_Con UTC Wet

100.00 43.40

Standard errors of differences of means

Table Res_Con

rep. 4

d.f. 9

s.e.d. 5.386

Least significant differences of means (5% level)

Table Res_Con

rep. 4

d.f. 9

l.s.d. 12.185

Stratum standard errors and coefficients of variation

Variate: SL%_of_Ctrl

Stratum d.f. s.e. cv%

Rep 3 1.132 1.7

Rep.PlotNo 9 7.617 11.6

Analysis of variance (Tri-Res-AR)

Variate: SL%_of_Ctrl

Source of variation d.f. s.s. m.s. v.r. F pr.

Rep stratum 3 87.52 29.17 1.00

Rep.PlotNo stratum

Res_Con 3 29007.27 9669.09 331.44 <.001

Residual 9 262.56 29.17

Total 15 29357.35

Tables of means

Variate: SL%_of_Ctrl

Grand mean 26.4

Res_Con Dry TC

5.4 0.0

Res_Con UTC Wet

100.0 0.0

Standard errors of differences of means

Table Res_Con

rep. 4

d.f. 9

s.e.d. 3.82

Least significant differences of means (5% level)

Table Res_Con

rep. 4

d.f. 9

l.s.d. 8.64

Stratum standard errors and coefficients of variation

Variate: SL%_of_Ctrl

Stratum d.f. s.e. cv%

Rep 3 2.70 10.2

Rep.PlotNo 9 5.40 20.5

Analysis of variance (Tri-Res-CU)

Variate: SL%_of_Ctrl

Source of variation d.f. s.s. m.s. v.r. F pr.

Rep stratum 3 43.46 14.49 0.81

Rep.PlotNo stratum

Res_Con 3 6391.99 2130.66 118.97 <.001

Residual 9 161.18 17.91

Total 15 6596.64

Tables of means

Variate: SL%_of_Ctrl

Grand mean 71.0

Res_Con Dry TC

77.0 46.1

Res_Con UTC Wet

100.0 60.9

Standard errors of differences of means

Table Res_Con

rep. 4

d.f. 9

s.e.d. 2.99

Least significant differences of means (5% level)

Table Res_Con

rep. 4

d.f. 9

l.s.d. 6.77

Stratum standard errors and coefficients of variation

Variate: SL%_of_Ctrl

Stratum d.f. s.e. cv%

Rep 3 1.90 2.7

Rep.PlotNo 9 4.23 6.0

Analysis of variance (Tri-Soil-AR)

Variate: SL%_of_Ctrl

Source of variation d.f. s.s. m.s. v.r. F pr.

Rep stratum 3 280.9 93.6 0.49

Rep.PlotNo stratum

Res_Con 3 10207.8 3402.6 17.98 <.001

Residual 9 1703.6 189.3

Total 15 12192.2

Tables of means

Variate: SL%_of_Ctrl

Grand mean 57.59

Res_Con Dry TC

33.35 48.01

Res_Con UTC Wet

100.00 48.98

Standard errors of differences of means

Table Res_Con

rep. 4

d.f. 9

s.e.d. 9.728

Least significant differences of means (5% level)

Table Res_Con

rep. 4

d.f. 9

l.s.d. 22.007

Stratum standard errors and coefficients of variation

Variate: SL%_of_Ctrl

Stratum d.f. s.e. cv%

Rep 3 4.838 8.4

Rep.PlotNo 9 13.758 23.9

Analysis of variance (Tri-Soil-CU)

Variate: SL%_of_Ctrl

Source of variation d.f. s.s. m.s. v.r. F pr.

Rep stratum 3 420.67 140.22 1.43

Rep.PlotNo stratum

Res_Con 3 3869.04 1289.68 13.18 0.001

Residual 9 880.98 97.89

Total 15 5170.69

Tables of means

Variate: SL%_of_Ctrl

Grand mean 78.93

Res_Con Dry TC

57.26 84.41

Res_Con UTC Wet

100.00 74.08

Standard errors of differences of means

Table Res_Con

rep. 4

d.f. 9

s.e.d. 6.996

Least significant differences of means (5% level)

Table Res_Con

rep. 4

d.f. 9

l.s.d. 15.826

Stratum standard errors and coefficients of variation

Variate: SL%_of_Ctrl

Stratum d.f. s.e. cv%

Rep 3 5.921 7.5

Rep.PlotNo 9 9.894 12.5
