# Supplementary material for: Effect of crop residues on interception and activity of prosulfocarb, pyroxasulfone, and trifluralin
Source: PLoS One. 2018 Dec 6;13(12):e0208274. doi: 10.1371/journal.pone.0208274 (PMC6283640; doi:10.1371/journal.pone.0208274)
Supplement: S3 Text — (DOCX) [file pone.0208274.s009.docx]

**Each herbicide with each plant species considered one experiment**:

744 "General Analysis of Variance."
 745 BLOCK Rep/PlotNo
 746 TREATMENTS Treat*R_Age*R_Type
 747 COVARIATE "No Covariate"
 748 ANOVA [PRINT=aovtable,information,means,%cv; FACT=32; CONTRASTS=7; PCONTRASTS=7; FPROB=yes;\
 749 PSE=diff,lsd; LSDLEVEL=5] SL%_of_Ctrl

Analysis of variance (Pro-Res-AR)

Variate: SL%_of_Ctrl

Source of variation d.f. s.s. m.s. v.r. F pr.

Rep stratum 3 53.727 17.909 1.99

Rep.PlotNo stratum

Treat 1 49.153 49.153 5.45 0.023

R_Age 1 23.440 23.440 2.60 0.112

R_Type 4 65.181 16.295 1.81 0.140

Treat.R_Age 1 11.236 11.236 1.25 0.269

Treat.R_Type 4 207.270 51.817 5.75 <.001

R_Age.R_Type 4 62.029 15.507 1.72 0.158

Treat.R_Age.R_Type 4 114.164 28.541 3.17 0.020

Residual 57 513.958 9.017

Total 79 1100.158

Tables of means

Variate: SL%_of_Ctrl

Grand mean 2.

Treat Rainfall TC UTC

2. 1.

R_Age Aged Fresh

1. 2.

R_Type Barley R.

0.

R_Type Canola R.

2.

R_Type Chickpea R.

3.

R_Type Lupin R.

1.

R_Type Wheat R.

1.

Treat R_Age Aged Fresh

Rainfall 2. 3.

TC 0. 2.

Treat R_Type Barley R.

Rainfall 0.

TC 1.

Treat R_Type Canola R.

Rainfall 3.

TC 1.

Treat R_Type Chickpea R.

Rainfall 7.

TC 0.

Treat R_Type Lupin R.

Rainfall 3.

TC 0.

Treat R_Type Wheat R.

Rainfall 0.

TC 3.

*Warning 2, code IO 56, statement 1 on line 545*

Command: ANOVA [PRINT=aovtable,information,means,%cv; FACT=32; CONTRASTS=7; PCON

Factor names or labels too wide to fit table across page.

*Warning 3, code IO 56, statement 1 on line 545*

Command: ANOVA [PRINT=aovtable,information,means,%cv; FACT=32; CONTRASTS=7; PCON

Factor names or labels too wide to fit table across page.

Standard errors of differences of means

Table Treat R_Age R_Type Treat

R_Age

rep. 40 40 16 20

d.f. 57 57 57 57

s.e.d. 0.7 0.7 1.1 0.9

Table Treat R_Age Treat

R_Type R_Type R_Age

R_Type

rep. 8 8 4

d.f. 57 57 57

s.e.d. 1.5 1.5 2.1

Least significant differences of means (5% level)

Table Treat R_Age R_Type Treat

R_Age

rep. 40 40 16 20

d.f. 57 57 57 57

l.s.d. 1.3 1.3 2.1 1.9

Table Treat R_Age Treat

R_Type R_Type R_Age

R_Type

rep. 8 8 4

d.f. 57 57 57

l.s.d. 3.0 3.0 4.3

Stratum standard errors and coefficients of variation

Variate: SL%_of_Ctrl

Stratum d.f. s.e. cv%

Rep 3 0.9 55.7

Rep.PlotNo 57 3.0 176.6

546 APLOT [RMETHOD=simple] fitted,normal,halfnormal,histogram

Analysis of variance (Pro-Res-CU)

Variate: SL%_of_Ctrl

Source of variation d.f. s.s. m.s. v.r. F pr.

Rep stratum 3 501.94 167.31 3.35

Rep.PlotNo stratum

Treat 1 3624.90 3624.90 72.55 <.001

R_Age 1 183.41 183.41 3.67 0.060

R_Type 4 7958.47 1989.62 39.82 <.001

Treat.R_Age 1 233.01 233.01 4.66 0.035

Treat.R_Type 4 2796.39 699.10 13.99 <.001

R_Age.R_Type 4 2896.92 724.23 14.50 <.001

Treat.R_Age.R_Type 4 657.82 164.46 3.29 0.017

Residual 57 2847.92 49.96

Total 79 21700.79

Tables of means

Variate: SL%_of_Ctrl

Grand mean 46.

Treat Rainfall TC UTC

53. 39.

R_Age Aged Fresh

44. 47.

R_Type Barley R.

57.

R_Type Canola R.

52.

R_Type Chickpea R.

46.

R_Type Lupin R.

27.

R_Type Wheat R.

47.

Treat R_Age Aged Fresh

Rainfall 49. 56.

TC 39. 39.

Treat R_Type Barley R.

Rainfall 61.

TC 52.

Treat R_Type Canola R.

Rainfall 55.

TC 49.

Treat R_Type Chickpea R.

Rainfall 64.

TC 27.

Treat R_Type Lupin R.

Rainfall 31.

TC 23.

Treat R_Type Wheat R.

Rainfall 51.

TC 44.

*Warning 4, code IO 56, statement 1 on line 604*

Command: ANOVA [PRINT=aovtable,information,means,%cv; FACT=32; CONTRASTS=7; PCON

Factor names or labels too wide to fit table across page.

*Warning 5, code IO 56, statement 1 on line 604*

Command: ANOVA [PRINT=aovtable,information,means,%cv; FACT=32; CONTRASTS=7; PCON

Factor names or labels too wide to fit table across page.

Standard errors of differences of means

Table Treat R_Age R_Type Treat

R_Age

rep. 40 40 16 20

d.f. 57 57 57 57

s.e.d. 1.6 1.6 2.5 2.2

Table Treat R_Age Treat

R_Type R_Type R_Age

R_Type

rep. 8 8 4

d.f. 57 57 57

s.e.d. 3.5 3.5 5.0

Least significant differences of means (5% level)

Table Treat R_Age R_Type Treat

R_Age

rep. 40 40 16 20

d.f. 57 57 57 57

l.s.d. 3.2 3.2 5.0 4.5

Table Treat R_Age Treat

R_Type R_Type R_Age

R_Type

rep. 8 8 4

d.f. 57 57 57

l.s.d. 7.1 7.1 10.0

Stratum standard errors and coefficients of variation

Variate: SL%_of_Ctrl

Stratum d.f. s.e. cv%

Rep 3 2.9 6.3

Rep.PlotNo 57 7.1 15.4

605 APLOT [RMETHOD=simple] fitted,normal,halfnormal,histogram

Analysis of variance (Pro-Soil-AR)

Variate: SL%_of_Ctrl

Source of variation d.f. s.s. m.s. v.r. F pr.

Rep stratum 3 3351. 1117. 0.74

Rep.PlotNo stratum

Treat 1 23464. 23464. 15.49 <.001

R_Age 1 824. 824. 0.54 0.464

R_Type 4 49443. 12361. 8.16 <.001

Treat.R_Age 1 5804. 5804. 3.83 0.055

Treat.R_Type 4 16774. 4194. 2.77 0.036

R_Age.R_Type 4 8019. 2005. 1.32 0.272

Treat.R_Age.R_Type 4 2920. 730. 0.48 0.749

Residual 57 86367. 1515.

Total 79 196967.

Tables of means

Variate: SL%_of_Ctrl

Grand mean 41.

Treat Rain TC UTC

23. 58.

R_Age A F

37. 44.

R_Type BR ChR

78. 16.

R_Type CR LR

35. 14.

R_Type WR

59.

Treat R_Age A F

Rain 12. 35.

TC 63. 52.

Treat R_Type BR

Rain 56.

TC 101.

Treat R_Type ChR

Rain 11.

TC 22.

Treat R_Type CR

Rain 29.

TC 41.

Treat R_Type LR

Rain 6.

TC 22.

Treat R_Type WR

Rain 16.

TC 102.

*Warning 6, code IO 56, statement 1 on line 577*

Command: ANOVA [PRINT=aovtable,information,means,%cv; FACT=32; CONTRASTS=7; PCON

Factor names or labels too wide to fit table across page.

*Warning 7, code IO 56, statement 1 on line 577*

Command: ANOVA [PRINT=aovtable,information,means,%cv; FACT=32; CONTRASTS=7; PCON

Factor names or labels too wide to fit table across page.

Standard errors of differences of means

Table Treat R_Age R_Type Treat

R_Age

rep. 40 40 16 20

d.f. 57 57 57 57

s.e.d. 8.7 8.7 13.8 12.3

Table Treat R_Age Treat

R_Type R_Type R_Age

R_Type

rep. 8 8 4

d.f. 57 57 57

s.e.d. 19.5 19.5 27.5

Least significant differences of means (5% level)

Table Treat R_Age R_Type Treat

R_Age

rep. 40 40 16 20

d.f. 57 57 57 57

l.s.d. 17.4 17.4 27.6 24.6

Table Treat R_Age Treat

R_Type R_Type R_Age

R_Type

rep. 8 8 4

d.f. 57 57 57

l.s.d. 39.0 39.0 55.1

Stratum standard errors and coefficients of variation

Variate: SL%_of_Ctrl

Stratum d.f. s.e. cv%

Rep 3 7.5 18.4

Rep.PlotNo 57 38.9 95.9

578 APLOT [RMETHOD=simple] fitted,normal,halfnormal,histogram

Analysis of variance (Pro-Soil-CU)

Variate: SL%_of_Ctrl

Source of variation d.f. s.s. m.s. v.r. F pr.

Rep stratum 3 125.49 41.83 0.80

Rep.PlotNo stratum

Treat 1 950.79 950.79 18.12 <.001

R_Age 1 119.04 119.04 2.27 0.138

R_Type 4 5392.37 1348.09 25.70 <.001

Treat.R_Age 1 0.40 0.40 0.01 0.931

Treat.R_Type 4 154.24 38.56 0.73 0.572

R_Age.R_Type 4 974.12 243.53 4.64 0.003

Treat.R_Age.R_Type 4 414.46 103.61 1.97 0.111

Residual 57 2990.43 52.46

Total 79 11121.35

*Message: the following units have large residuals.*

Rep 2 PlotNo 18 -30. approx. s.e. 6.

Tables of means

Variate: SL%_of_Ctrl

Grand mean 27.

Treat Rain TC UTC

24. 31.

R_Age A F

26. 29.

R_Type BR ChR

37. 18.

R_Type CR LR

25. 20.

R_Type WR

37.

Treat R_Age A F

Rain 23. 25.

TC 30. 32.

Treat R_Type BR

Rain 34.

TC 39.

Treat R_Type ChR

Rain 15.

TC 20.

Treat R_Type CR

Rain 19.

TC 31.

Treat R_Type LR

Rain 16.

TC 24.

Treat R_Type WR

Rain 34.

TC 40.

*Warning 8, code IO 56, statement 1 on line 610*

Command: ANOVA [PRINT=aovtable,information,means,%cv; FACT=32; CONTRASTS=7; PCON

Factor names or labels too wide to fit table across page.

*Warning 9, code IO 56, statement 1 on line 610*

Command: ANOVA [PRINT=aovtable,information,means,%cv; FACT=32; CONTRASTS=7; PCON

Factor names or labels too wide to fit table across page.

Standard errors of differences of means

Table Treat R_Age R_Type Treat

R_Age

rep. 40 40 16 20

d.f. 57 57 57 57

s.e.d. 1.6 1.6 2.6 2.3

Table Treat R_Age Treat

R_Type R_Type R_Age

R_Type

rep. 8 8 4

d.f. 57 57 57

s.e.d. 3.6 3.6 5.1

Least significant differences of means (5% level)

Table Treat R_Age R_Type Treat

R_Age

rep. 40 40 16 20

d.f. 57 57 57 57

l.s.d. 3.2 3.2 5.1 4.6

Table Treat R_Age Treat

R_Type R_Type R_Age

R_Type

rep. 8 8 4

d.f. 57 57 57

l.s.d. 7.3 7.3 10.3

Stratum standard errors and coefficients of variation

Variate: SL%_of_Ctrl

Stratum d.f. s.e. cv%

Rep 3 1.4 5.3

Rep.PlotNo 57 7.2 26.5

Analysis of variance (Pyro-Res-AR)

Variate: SL%_of_Ctrl

Source of variation d.f. s.s. m.s. v.r. F pr.

Rep stratum 3 54.25 18.08 1.21

Rep.PlotNo stratum

Treat 1 161.98 161.98 10.83 0.002

R_Age 1 0.36 0.36 0.02 0.877

R_Type 4 508.69 127.17 8.50 <.001

Treat.R_Age 1 0.36 0.36 0.02 0.877

Treat.R_Type 4 508.69 127.17 8.50 <.001

R_Age.R_Type 4 23.32 5.83 0.39 0.815

Treat.R_Age.R_Type 4 23.32 5.83 0.39 0.815

Residual 57 852.81 14.96

Total 79 2133.78

Tables of means

Variate: SL%_of_Ctrl

Grand mean 1.

Treat Rainfall TC UTC

3. 0.

R_Age Aged Fresh

1. 1.

R_Type Barley R.

0.

R_Type Canola R.

1.

R_Type Chickpea R.

0.

R_Type Lupin R.

6.

R_Type Wheat R.

0.

Treat R_Age Aged Fresh

Rainfall 3. 3.

TC 0. 0.

Treat R_Type Barley R.

Rainfall 0.

TC 0.

Treat R_Type Canola R.

Rainfall 1.

TC 0.

Treat R_Type Chickpea R.

Rainfall 0.

TC 0.

Treat R_Type Lupin R.

Rainfall 13.

TC 0.

Treat R_Type Wheat R.

Rainfall 0.

TC 0.

*Warning 6, code IO 56, statement 1 on line 663*

Command: ANOVA [PRINT=aovtable,information,means,%cv; FACT=32; CONTRASTS=7; PCON

Factor names or labels too wide to fit table across page.

*Warning 7, code IO 56, statement 1 on line 663*

Command: ANOVA [PRINT=aovtable,information,means,%cv; FACT=32; CONTRASTS=7; PCON

Factor names or labels too wide to fit table across page.

Standard errors of differences of means

Table Treat R_Age R_Type Treat

R_Age

rep. 40 40 16 20

d.f. 57 57 57 57

s.e.d. 0.9 0.9 1.4 1.2

Table Treat R_Age Treat

R_Type R_Type R_Age

R_Type

rep. 8 8 4

d.f. 57 57 57

s.e.d. 1.9 1.9 2.7

Least significant differences of means (5% level)

Table Treat R_Age R_Type Treat

R_Age

rep. 40 40 16 20

d.f. 57 57 57 57

l.s.d. 1.7 1.7 2.7 2.4

Table Treat R_Age Treat

R_Type R_Type R_Age

R_Type

rep. 8 8 4

d.f. 57 57 57

l.s.d. 3.9 3.9 5.5

Stratum standard errors and coefficients of variation

Variate: SL%_of_Ctrl

Stratum d.f. s.e. cv%

Rep 3 1.0 66.8

Rep.PlotNo 57 3.9 271.8

664 APLOT [RMETHOD=simple] fitted,normal,halfnormal,histogram

Analysis of variance (Pyro-Res-CU)

Variate: SL%_of_Ctrl

Source of variation d.f. s.s. m.s. v.r. F pr.

Rep stratum 3 188.91 62.97 1.05

Rep.PlotNo stratum

Treat 1 2857.75 2857.75 47.77 <.001

R_Age 1 1268.53 1268.53 21.21 <.001

R_Type 4 14537.71 3634.43 60.76 <.001

Treat.R_Age 1 646.71 646.71 10.81 0.002

Treat.R_Type 4 1486.02 371.51 6.21 <.001

R_Age.R_Type 4 3027.18 756.79 12.65 <.001

Treat.R_Age.R_Type 4 361.09 90.27 1.51 0.212

Residual 57 3409.68 59.82

Total 79 27783.59

Tables of means

Variate: SL%_of_Ctrl

Grand mean 53.

Treat Rainfall TC UTC

59. 47.

R_Age Aged Fresh

57. 49.

R_Type Barley R.

72.

R_Type Canola R.

52.

R_Type Chickpea R.

57.

R_Type Lupin R.

30.

R_Type Wheat R.

55.

Treat R_Age Aged Fresh

Rainfall 60. 58.

TC 54. 40.

Treat R_Type Barley R.

Rainfall 73.

TC 72.

Treat R_Type Canola R.

Rainfall 59.

TC 45.

Treat R_Type Chickpea R.

Rainfall 70.

TC 43.

Treat R_Type Lupin R.

Rainfall 35.

TC 26.

Treat R_Type Wheat R.

Rainfall 59.

TC 51.

*Warning 9, code IO 56, statement 1 on line 696*

Command: ANOVA [PRINT=aovtable,information,means,%cv; FACT=32; CONTRASTS=7; PCON

Factor names or labels too wide to fit table across page.

*Warning 10, code IO 56, statement 1 on line 696*

Command: ANOVA [PRINT=aovtable,information,means,%cv; FACT=32; CONTRASTS=7; PCON

Factor names or labels too wide to fit table across page.

Standard errors of differences of means

Table Treat R_Age R_Type Treat

R_Age

rep. 40 40 16 20

d.f. 57 57 57 57

s.e.d. 1.7 1.7 2.7 2.4

Table Treat R_Age Treat

R_Type R_Type R_Age

R_Type

rep. 8 8 4

d.f. 57 57 57

s.e.d. 3.9 3.9 5.5

Least significant differences of means (5% level)

Table Treat R_Age R_Type Treat

R_Age

rep. 40 40 16 20

d.f. 57 57 57 57

l.s.d. 3.5 3.5 5.5 4.9

Table Treat R_Age Treat

R_Type R_Type R_Age

R_Type

rep. 8 8 4

d.f. 57 57 57

l.s.d. 7.7 7.7 11.0

Stratum standard errors and coefficients of variation

Variate: SL%_of_Ctrl

Stratum d.f. s.e. cv%

Rep 3 1.8 3.3

Rep.PlotNo 57 7.7 14.5

697 APLOT [RMETHOD=simple] fitted,normal,halfnormal,histogram

Analysis of variance (Pyro-Soil-AR)

Variate: SL%_of_Ctrl

Source of variation d.f. s.s. m.s. v.r. F pr.

Rep stratum 3 209.0 69.7 0.34

Rep.PlotNo stratum

Treat 1 7436.4 7436.4 36.30 <.001

R_Age 1 31.8 31.8 0.16 0.695

R_Type 4 10488.0 2622.0 12.80 <.001

Treat.R_Age 1 31.8 31.8 0.16 0.695

Treat.R_Type 4 10488.0 2622.0 12.80 <.001

R_Age.R_Type 4 43.3 10.8 0.05 0.995

Treat.R_Age.R_Type 4 43.3 10.8 0.05 0.995

Residual 57 11675.9 204.8

Total 79 40447.3

Tables of means

Variate: SL%_of_Ctrl

Grand mean 10.

Treat Rain TC UTC

0. 19.

R_Age A F

9. 10.

R_Type BR ChR

28. 0.

R_Type CR LR

2. 0.

R_Type WR

19.

Treat R_Age A F

Rain 0. 0.

TC 18. 21.

Treat R_Type BR

Rain 0.

TC 55.

Treat R_Type ChR

Rain 0.

TC 0.

Treat R_Type CR

Rain 0.

TC 3.

Treat R_Type LR

Rain 0.

TC 0.

Treat R_Type WR

Rain 0.

TC 38.

*Warning 13, code IO 56, statement 1 on line 650*

Command: ANOVA [PRINT=aovtable,information,means,%cv; FACT=32; CONTRASTS=7; PCON

Factor names or labels too wide to fit table across page.

*Warning 14, code IO 56, statement 1 on line 650*

Command: ANOVA [PRINT=aovtable,information,means,%cv; FACT=32; CONTRASTS=7; PCON

Factor names or labels too wide to fit table across page.

Standard errors of differences of means

Table Treat R_Age R_Type Treat

R_Age

rep. 40 40 16 20

d.f. 57 57 57 57

s.e.d. 3.2 3.2 5.1 4.5

Table Treat R_Age Treat

R_Type R_Type R_Age

R_Type

rep. 8 8 4

d.f. 57 57 57

s.e.d. 7.2 7.2 10.1

Least significant differences of means (5% level)

Table Treat R_Age R_Type Treat

R_Age

rep. 40 40 16 20

d.f. 57 57 57 57

l.s.d. 6.4 6.4 10.1 9.1

Table Treat R_Age Treat

R_Type R_Type R_Age

R_Type

rep. 8 8 4

d.f. 57 57 57

l.s.d. 14.3 14.3 20.3

Stratum standard errors and coefficients of variation

Variate: SL%_of_Ctrl

Stratum d.f. s.e. cv%

Rep 3 1.9 19.4

Rep.PlotNo 57 14.3 148.4

651 APLOT [RMETHOD=simple] fitted,normal,halfnormal,histogram

Analysis of variance (Pyro-Soil-CU)

Variate: SL%_of_Ctrl

Source of variation d.f. s.s. m.s. v.r. F pr.

Rep stratum 3 131.6 43.9 0.76

Rep.PlotNo stratum

Treat 1 3866.8 3866.8 66.87 <.001

R_Age 1 10848.7 10848.7 187.62 <.001

R_Type 4 31669.3 7917.3 136.92 <.001

Treat.R_Age 1 108.4 108.4 1.88 0.176

Treat.R_Type 4 379.4 94.9 1.64 0.177

R_Age.R_Type 4 3213.4 803.4 13.89 <.001

Treat.R_Age.R_Type 4 1652.8 413.2 7.15 <.001

Residual 57 3295.9 57.8 0.12

Rep.PlotNo.*Units* stratum
 160 75124.8 469.5

Total 239 130291.0

Tables of means

Variate: SL%_of_Ctrl

Grand mean 40.

Treat Rain TC UTC

36. 44.

R_Age A F

33. 46.

R_Type BR ChR

55. 27.

R_Type CR LR

37. 28.

R_Type WR

51.

Treat R_Age A F

Rain 30. 42.

TC 36. 51.

Treat R_Type BR

Rain 51.

TC 60.

Treat R_Type ChR

Rain 26.

TC 29.

Treat R_Type CR

Rain 31.

TC 42.

Treat R_Type LR

Rain 24.

TC 32.

Treat R_Type WR

Rain 47.

TC 55.

*Warning 16, code IO 56, statement 1 on line 683*

Command: ANOVA [PRINT=aovtable,information,means,%cv; FACT=32; CONTRASTS=7; PCON

Factor names or labels too wide to fit table across page.

*Warning 17, code IO 56, statement 1 on line 683*

Command: ANOVA [PRINT=aovtable,information,means,%cv; FACT=32; CONTRASTS=7; PCON

Factor names or labels too wide to fit table across page.

Standard errors of differences of means

Table Treat R_Age R_Type Treat

R_Age

rep. 120 120 48 60

d.f. 57 57 57 57

s.e.d. 1.0 1.0 1.6 1.4

Table Treat R_Age Treat

R_Type R_Type R_Age

R_Type

rep. 24 24 12

d.f. 57 57 57

s.e.d. 2.2 2.2 3.1

Least significant differences of means (5% level)

Table Treat R_Age R_Type Treat

R_Age

rep. 120 120 48 60

d.f. 57 57 57 57

l.s.d. 2.0 2.0 3.1 2.8

Table Treat R_Age Treat

R_Type R_Type R_Age

R_Type

rep. 24 24 12

d.f. 57 57 57

l.s.d. 4.4 4.4 6.2

Stratum standard errors and coefficients of variation

Variate: SL%_of_Ctrl

Stratum d.f. s.e. cv%

Rep 3 0.9 2.2

Rep.PlotNo 57 4.4 11.1

Rep.PlotNo.*Units* 160 21.7 54.7

684 APLOT [RMETHOD=simple] fitted,normal,halfnormal,histogram

Analysis of variance (Tri-Res-AR)

Variate: SL%_of_Ctrl

Source of variation d.f. s.s. m.s. v.r. F pr.

Rep stratum 3 81.61 27.20 0.36

Rep.PlotNo stratum

Treat 1 2173.52 2173.52 28.99 <.001

R_Age 1 5.76 5.76 0.08 0.783

R_Type 4 5730.83 1432.71 19.11 <.001

Treat.R_Age 1 18.41 18.41 0.25 0.622

Treat.R_Type 4 4916.42 1229.10 16.39 <.001

R_Age.R_Type 4 554.38 138.59 1.85 0.132

Treat.R_Age.R_Type 4 934.12 233.53 3.11 0.022

Residual 57 4274.24 74.99

Total 79 18689.28

Tables of means

Variate: SL%_of_Ctrl

Grand mean 8.

Treat Rainfall TC UTC

13. 3.

R_Age Aged Fresh

8. 8.

R_Type Barley R.

0.

R_Type Canola R.

3.

R_Type Chickpea R.

22.

R_Type Lupin R.

14.

R_Type Wheat R.

1.

Treat R_Age Aged Fresh

Rainfall 12. 14.

TC 3. 3.

Treat R_Type Barley R.

Rainfall 0.

TC 0.

Treat R_Type Canola R.

Rainfall 5.

TC 1.

Treat R_Type Chickpea R.

Rainfall 42.

TC 2.

Treat R_Type Lupin R.

Rainfall 19.

TC 8.

Treat R_Type Wheat R.

Rainfall 0.

TC 3.

*Warning 12, code IO 56, statement 1 on line 729*

Command: ANOVA [PRINT=aovtable,information,means,%cv; FACT=32; CONTRASTS=7; PCON

Factor names or labels too wide to fit table across page.

*Warning 13, code IO 56, statement 1 on line 729*

Command: ANOVA [PRINT=aovtable,information,means,%cv; FACT=32; CONTRASTS=7; PCON

Factor names or labels too wide to fit table across page.

Standard errors of differences of means

Table Treat R_Age R_Type Treat

R_Age

rep. 40 40 16 20

d.f. 57 57 57 57

s.e.d. 1.9 1.9 3.1 2.7

Table Treat R_Age Treat

R_Type R_Type R_Age

R_Type

rep. 8 8 4

d.f. 57 57 57

s.e.d. 4.3 4.3 6.1

Least significant differences of means (5% level)

Table Treat R_Age R_Type Treat

R_Age

rep. 40 40 16 20

d.f. 57 57 57 57

l.s.d. 3.9 3.9 6.1 5.5

Table Treat R_Age Treat

R_Type R_Type R_Age

R_Type

rep. 8 8 4

d.f. 57 57 57

l.s.d. 8.7 8.7 12.3

Stratum standard errors and coefficients of variation

Variate: SL%_of_Ctrl

Stratum d.f. s.e. cv%

Rep 3 1.2 14.5

Rep.PlotNo 57 8.7 107.9

730 APLOT [RMETHOD=simple] fitted,normal,halfnormal,histogram

Analysis of variance (Tri-Res-CU)

Variate: SL%_of_Ctrl

Source of variation d.f. s.s. m.s. v.r. F pr.

Rep stratum 3 709.65 236.55 5.34

Rep.PlotNo stratum

Treat 1 3151.27 3151.27 71.20 <.001

R_Age 1 27.95 27.95 0.63 0.430

R_Type 4 11543.39 2885.85 65.21 <.001

Treat.R_Age 1 79.14 79.14 1.79 0.186

Treat.R_Type 4 797.19 199.30 4.50 0.003

R_Age.R_Type 4 4948.15 1237.04 27.95 <.001

Treat.R_Age.R_Type 4 352.77 88.19 1.99 0.108

Residual 57 2522.66 44.26

Total 79 24132.16

Tables of means

Variate: SL%_of_Ctrl

Grand mean 52.

Treat Rainfall TC UTC

58. 46.

R_Age Aged Fresh

51. 52.

R_Type Barley R.

62.

R_Type Canola R.

47.

R_Type Chickpea R.

58.

R_Type Lupin R.

30.

R_Type Wheat R.

61.

Treat R_Age Aged Fresh

Rainfall 57. 60.

TC 46. 45.

Treat R_Type Barley R.

Rainfall 72.

TC 53.

Treat R_Type Canola R.

Rainfall 48.

TC 47.

Treat R_Type Chickpea R.

Rainfall 65.

TC 51.

Treat R_Type Lupin R.

Rainfall 39.

TC 22.

Treat R_Type Wheat R.

Rainfall 68.

TC 55.

*Warning 15, code IO 56, statement 1 on line 762*

Command: ANOVA [PRINT=aovtable,information,means,%cv; FACT=32; CONTRASTS=7; PCON

Factor names or labels too wide to fit table across page.

*Warning 16, code IO 56, statement 1 on line 762*

Command: ANOVA [PRINT=aovtable,information,means,%cv; FACT=32; CONTRASTS=7; PCON

Factor names or labels too wide to fit table across page.

Standard errors of differences of means

Table Treat R_Age R_Type Treat

R_Age

rep. 40 40 16 20

d.f. 57 57 57 57

s.e.d. 1.5 1.5 2.4 2.1

Table Treat R_Age Treat

R_Type R_Type R_Age

R_Type

rep. 8 8 4

d.f. 57 57 57

s.e.d. 3.3 3.3 4.7

Least significant differences of means (5% level)

Table Treat R_Age R_Type Treat

R_Age

rep. 40 40 16 20

d.f. 57 57 57 57

l.s.d. 3.0 3.0 4.7 4.2

Table Treat R_Age Treat

R_Type R_Type R_Age

R_Type

rep. 8 8 4

d.f. 57 57 57

l.s.d. 6.7 6.7 9.4

Stratum standard errors and coefficients of variation

Variate: SL%_of_Ctrl

Stratum d.f. s.e. cv%

Rep 3 3.4 6.6

Rep.PlotNo 57 6.7 12.8

763 APLOT [RMETHOD=simple] fitted,normal,halfnormal,histogram

Analysis of variance (Tri-Soil-AR)

Variate: SL%_of_Ctrl

Source of variation d.f. s.s. m.s. v.r. F pr.

Rep stratum 3 1215.7 405.2 1.45

Rep.PlotNo stratum

Treat 1 8549.0 8549.0 30.69 <.001

R_Age 1 3811.7 3811.7 13.68 <.001

R_Type 4 20531.5 5132.9 18.43 <.001

Treat.R_Age 1 426.1 426.1 1.53 0.221

Treat.R_Type 4 271.8 68.0 0.24 0.912

R_Age.R_Type 4 1414.3 353.6 1.27 0.293

Treat.R_Age.R_Type 4 3843.1 960.8 3.45 0.014

Residual 57 15878.7 278.6

Total 79 55942.0

Tables of means

Variate: SL%_of_Ctrl

Grand mean 32.

Treat Rain TC UTC

22. 43.

R_Age A F

25. 39.

R_Type BR ChR

56. 19.

R_Type CR LR

18. 21.

R_Type WR

47.

Treat R_Age A F

Rain 17. 27.

TC 33. 52.

Treat R_Type BR

Rain 46.

TC 66.

Treat R_Type ChR

Rain 6.

TC 32.

Treat R_Type CR

Rain 8.

TC 29.

Treat R_Type LR

Rain 11.

TC 32.

Treat R_Type WR

Rain 39.

TC 54.

*Warning 19, code IO 56, statement 1 on line 716*

Command: ANOVA [PRINT=aovtable,information,means,%cv; FACT=32; CONTRASTS=7; PCON

Factor names or labels too wide to fit table across page.

*Warning 20, code IO 56, statement 1 on line 716*

Command: ANOVA [PRINT=aovtable,information,means,%cv; FACT=32; CONTRASTS=7; PCON

Factor names or labels too wide to fit table across page.

Standard errors of differences of means

Table Treat R_Age R_Type Treat

R_Age

rep. 40 40 16 20

d.f. 57 57 57 57

s.e.d. 3.7 3.7 5.9 5.3

Table Treat R_Age Treat

R_Type R_Type R_Age

R_Type

rep. 8 8 4

d.f. 57 57 57

s.e.d. 8.3 8.3 11.8

Least significant differences of means (5% level)

Table Treat R_Age R_Type Treat

R_Age

rep. 40 40 16 20

d.f. 57 57 57 57

l.s.d. 7.5 7.5 11.8 10.6

Table Treat R_Age Treat

R_Type R_Type R_Age

R_Type

rep. 8 8 4

d.f. 57 57 57

l.s.d. 16.7 16.7 23.6

Stratum standard errors and coefficients of variation

Variate: SL%_of_Ctrl

Stratum d.f. s.e. cv%

Rep 3 4.5 13.9

Rep.PlotNo 57 16.7 51.7

717 APLOT [RMETHOD=simple] fitted,normal,halfnormal,histogram

Analysis of variance (Tri-Soil-CU)

Variate: SL%_of_Ctrl

Source of variation d.f. s.s. m.s. v.r. F pr.

Rep stratum 3 96.29 32.10 0.36

Rep.PlotNo stratum

Treat 1 856.69 856.69 9.70 0.003

R_Age 1 4967.94 4967.94 56.26 <.001

R_Type 4 28829.14 7207.28 81.62 <.001

Treat.R_Age 1 191.63 191.63 2.17 0.146

Treat.R_Type 4 894.33 223.58 2.53 0.050

R_Age.R_Type 4 4723.88 1180.97 13.37 <.001

Treat.R_Age.R_Type 4 3011.16 752.79 8.53 <.001

Residual 57 5033.04 88.30

Total 79 48604.09

Tables of means

Variate: SL%_of_Ctrl

Grand mean 59.

Treat Rain TC UTC

56. 63.

R_Age A F

51. 67.

R_Type BR ChR

86. 39.

R_Type CR LR

54. 41.

R_Type WR

77.

Treat R_Age A F

Rain 47. 65.

TC 56. 69.

Treat R_Type BR

Rain 82.

TC 90.

Treat R_Type ChR

Rain 41.

TC 38.

Treat R_Type CR

Rain 48.

TC 60.

Treat R_Type LR

Rain 33.

TC 48.

Treat R_Type WR

Rain 76.

TC 77.

*Warning 21, code IO 56, statement 1 on line 749*

Command: ANOVA [PRINT=aovtable,information,means,%cv; FACT=32; CONTRASTS=7; PCON

Factor names or labels too wide to fit table across page.

*Warning 22, code IO 56, statement 1 on line 749*

Command: ANOVA [PRINT=aovtable,information,means,%cv; FACT=32; CONTRASTS=7; PCON

Factor names or labels too wide to fit table across page.

Standard errors of differences of means

Table Treat R_Age R_Type Treat

R_Age

rep. 40 40 16 20

d.f. 57 57 57 57

s.e.d. 2.1 2.1 3.3 3.0

Table Treat R_Age Treat

R_Type R_Type R_Age

R_Type

rep. 8 8 4

d.f. 57 57 57

s.e.d. 4.7 4.7 6.6

Least significant differences of means (5% level)

Table Treat R_Age R_Type Treat

R_Age

rep. 40 40 16 20

d.f. 57 57 57 57

l.s.d. 4.2 4.2 6.7 6.0

Table Treat R_Age Treat

R_Type R_Type R_Age

R_Type

rep. 8 8 4

d.f. 57 57 57

l.s.d. 9.4 9.4 13.3

Stratum standard errors and coefficients of variation

Variate: SL%_of_Ctrl

Stratum d.f. s.e. cv%

Rep 3 1.3 2.1

Rep.PlotNo 57 9.4 15.8

750 APLOT [RMETHOD=simple] fitted,normal,halfnormal,histogram
